# Large-scale expansions of Friedreich's ataxia GAA•TTC repeats in an experimental human system: role of DNA replication and prevention by LNA-DNA oligonucleotides and PNA oligomers

**DOI:** 10.1093/nar/gkad441

**Published:** 2023-05-22

**Authors:** Anastasia Rastokina, Jorge Cebrián, Negin Mozafari, Nicholas H Mandel, C I Edvard Smith, Massimo Lopes, Rula Zain, Sergei M Mirkin

**Affiliations:** Department of Biology, Tufts University, Medford, MA 02155, USA; Department of Biology, Tufts University, Medford, MA 02155, USA; Department of Laboratory Medicine, Translational Research Center Karolinska (TRACK), Karolinska Institutet, Karolinska University Hospital, SE-171 77 Stockholm, Sweden; Department of Biology, Tufts University, Medford, MA 02155, USA; Department of Laboratory Medicine, Translational Research Center Karolinska (TRACK), Karolinska Institutet, Karolinska University Hospital, SE-171 77 Stockholm, Sweden; Institute of Molecular Cancer Research, University of Zurich, Zurich 8057, Switzerland; Department of Laboratory Medicine, Translational Research Center Karolinska (TRACK), Karolinska Institutet, Karolinska University Hospital, SE-171 77 Stockholm, Sweden; Center for Rare Diseases, Karolinska University Hospital, SE-17176 Stockholm, Sweden; Department of Biology, Tufts University, Medford, MA 02155, USA

## Abstract

Friedreich's ataxia (FRDA) is caused by expansions of GAA•TTC repeats in the first intron of the human *FXN* gene that occur during both intergenerational transmissions and in somatic cells. Here we describe an experimental system to analyze large-scale repeat expansions in cultured human cells. It employs a shuttle plasmid that can replicate from the SV40 origin in human cells or be stably maintained in *S. cerevisiae* utilizing ARS4-CEN6. It also contains a selectable cassette allowing us to detect repeat expansions that accumulated in human cells upon plasmid transformation into yeast. We indeed observed massive expansions of GAA•TTC repeats, making it the first genetically tractable experimental system to study large-scale repeat expansions in human cells. Further, GAA•TTC repeats stall replication fork progression, while the frequency of repeat expansions appears to depend on proteins implicated in replication fork stalling, reversal, and restart. Locked nucleic acid (LNA)-DNA mixmer oligonucleotides and peptide nucleic acid (PNA) oligomers, which interfere with triplex formation at GAA•TTC repeats *in vitro*, prevented the expansion of these repeats in human cells. We hypothesize, therefore, that triplex formation by GAA•TTC repeats stall replication fork progression, ultimately leading to repeat expansions during replication fork restart.

## Graphical Abstract

**Graphical Abstract:**
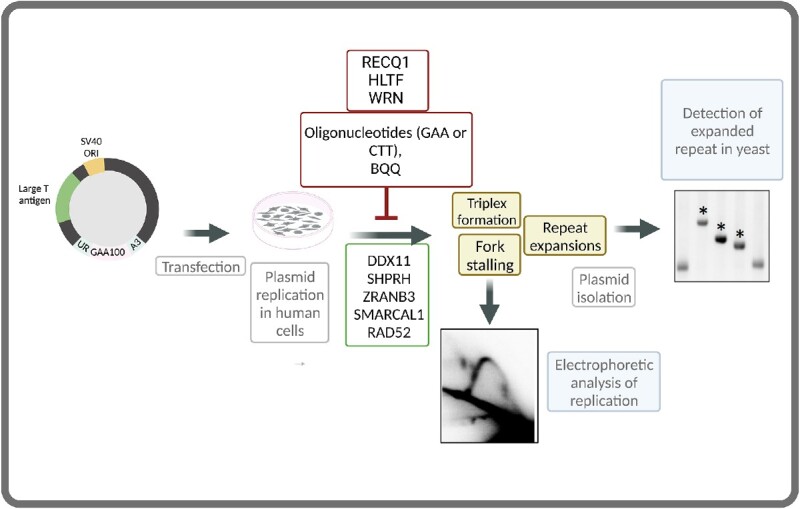

## INTRODUCTION

Expansions of simple, tandem DNA repeats cause over 50 hereditary diseases in humans (1–3). One of these diseases is Friedreich's ataxia (FRDA): a rare, autosomal recessive degenerative disease caused by the expansion of GAA•TTC repeats in the first intron of the frataxin gene (*FXN*) (4). Normal alleles contain from 7 to 22 repeats, while FRDA patients have 66 to 1700 repeats in both alleles of the *FXN* gene (5). Lengthening of GAA•TTC tracts results in a progressive reduction of the *FXN* mRNA (6–8), frataxin deficiency, mitochondrial dysfunction and cell death (9,10). Consequently, the lengths of GAA•TTC repeats correlate directly with the severity of the disease and inversely with its age-at-onset (11).

The nuclear *FXN* gene encodes the protein frataxin, which predominantly localizes in mitochondria, but is also weakly expressed in nuclei, endoplasmic reticulum and microsomes (12,13). It is responsible for iron-sulfur (Fe-S) cluster biosynthesis, thus, diminished levels of frataxin lead to toxic iron accumulation in mitochondria, elevation of cellular oxidative stress and subsequent cell death (14–16). Neurodegeneration in FRDA is characterized by damage in the spinal cord, dorsal root ganglia (DRG) and cerebellum (17,18). Patients often develop sensory and motor dysfunction at puberty and eventually lose their ability to walk. They also suffer from progressive cardiomyopathy, which is the leading cause of death (19).

*FXN* downregulation by expanded GAA•TTC repeats is triggered by the formation of an intramolecular DNA triplex, H-DNA, which impedes transcription elongation (20–22), ultimately leading to local heterochromatinization and gene silencing (23). Two types of triplex structures were shown to be formed by pathogenic GAA•TTC repeats *in vitro*: pyrimidine-purine-pyrimidine (H-y) or purine-purine-pyrimidine (H-r) where the third strand is homopyrimidine or homopurine, respectively (24–29). Another, less characterized structure called sticky-DNA involves a triplex formed by two distant GAA•TTC runs (30,31).

GAA•TTC repeats are unstable during germline transmission from parent to offspring, which can result in large-scale repeat expansions or contractions between generations (32,33). Pathogenic GAA•TTC repeats also expand further in somatic cells resulting in disease progression during the affected individual's lifespan (34–36). The most prominent expansions occur in DRG followed by the cerebellum and heart (37). It was hypothesized that formation of triplex DNA could be responsible for GAA•TTC repeat expansions in FRDA patients (38,39). One argument supporting this hypothesis is the fact that a GAAGGA•TCCTTC repeat, which cannot form an H-DNA structure, in the *FXN* gene is stable and results in a very mild and late-onset disease (40).

Mechanisms responsible for GAA•TTC repeat expansions were primarily studied in model experimental systems. First, these repeats were shown to block DNA polymerization *in vitro* (24,41) and stall replication fork progression in bacteria (35), yeast (42), mammalian cell culture (43,44) and patient-derived iPSC cells (45). Further, we have previously developed an experimental system to measure the rate of large-scale expansions of GAA•TTC repeats in yeast (46). A subsequent unbiassed, genome-wide genetic screen identified several dozens of genes affecting the rate of repeat expansions, most of which encoded replication fork components (47). Replicative DNA polymerases and proteins involved in Okazaki fragment maturation strongly counteract repeat expansions (48–50). Finally, stabilization of H-DNA formed by GAA•TTC repeats by an RNA transcript (H-loop) additionally increases repeat instability (51). Altogether, these data led us to propose that GAA•TTC repeat expansions in yeast occur while the replication fork struggles to progress through the structure-prone DNA element. Replication was also implicated in the stability of GAA•TTC repeats within the SV40-based mammalian episome (52).

Other machinery implicated in GAA•TTC repeat instability is DNA mismatch repair (MMR). In yeast, the MutLα complex appears to cleave H-DNA, which results in chromosomal fragility in dividing cells (53). Notably, most affected cells in FRDA are post-mitotic, thus, repeat instability in those cells is independent of DNA replication. In an experimental yeast system to study GAA•TTC repeat instability in non-dividing, quiescent cells, MMR counteracted repeat expansions by triggering the formation of deletions or gene conversion events (54). Contrasting results were obtained in a human cell line characterized by progressive, small-scale expansions of GAA•TTC repeats: They appear to be independent of cell division and promoted by the mismatch repair complex MutLγ (55). Similarly, MMR promoted GAA•TTC repeat expansions in iPSCs derived from FRDA patient fibroblasts (56). Expansions of GAA•TTC repeats were also studied in humanized mice. In this system, intergenerational expansions of GAA•TTC repeats were inhibited by a mismatch repair system, while somatic expansions in the cerebellum and DRG were promoted by MMR (57,58).

In this paper, we aimed to study the mechanisms of large-scale expansions of GAA•TTC repeats in human cells. To this end, we designed an experimental system that allowed us to simultaneously analyze DNA replication of GAA•TTC repeats and their large-scale expansions in cultured human cells. It is based on a shuttle plasmid that expresses T-antigen (Tag), driving its extremely efficient replication from the SV40 origin in human cells (43,44). It also contains our previously described cassette for selecting repeat expansions in yeast and can be stably maintained in *Saccharomyces cerevisiae* (46). Repeat expansions accumulated during replication of this vector in mammalian cells are then detected upon its transformation into yeast. A similar strategy has been successfully used in the Lahue lab to analyze mid-scale expansions of CAG•CTG repeats in human astrocytes (59–61). Our system differs in two significant regards: (1) The presence of Tag in the plasmid magnifies its replication in human cells, making the electrophoretic analysis of the replication fork progression feasible; and (2) Our selectable cassette is tuned for studying large-scale repeat expansions.

We confirmed that GAA•TTC repeats cause stalling and reversal of the replication fork in human cells. Remarkably, large-scale expansions of GAA•TTC repeats efficiently occur in this system, making it the first experimental model for human cells. We conducted a candidate gene analysis of large-scale repeat expansions using the siRNA-mediated gene silencing approach. The depletion of 8 of the 20 proteins tested significantly impacted the frequency of GAA•TTC repeat expansions. Notably, those proteins were previously implicated in the unwinding of triplex DNA, fork reversal, and fork restoration (62).

Using our in-house triplex-specific cleavage assay, we showed that H-DNA is efficiently formed by the GAA•TTC repeat in the supercoiled plasmid used for replication/expansion studies in human cells. We have previously reported that chemically modified oligonucleotides, which bind sequence-specifically to the GAA•TTC repeats, disrupt H-DNA formation in supercoiled plasmids (26). Here, we examined the ability of these oligonucleotides to affect the expansion of GAA•TTC repeats in human cells. We found that the LNA-DNA mixmer oligonucleotides, hereafter referred to as LNA-ONs, and the corresponding PNA oligomers dramatically reduce the expansion frequency of GAA•TTC repeats. These data hold promise for the development of these compounds for the treatment of FRDA, which is currently incurable.

Altogether, our data led us to hypothesize that triplex formation by GAA•TTC repeats impairs replication fork progression, ultimately leading to their expansions during replication fork restart.

## MATERIALS AND METHODS

### Plasmids

The plasmid pJC_GAA100 was constructed by conventional cloning methods in several steps using the pLM113 plasmid as a backbone (44). First, pRS316 (63) was digested by SalI and SacI to excise the ARS4-CEN6 module, which was inserted between SalI and SacI sites of the pML113 plasmid, creating a plasmid called pJW12 (8835 bp-long). The pJC_GAA100 plasmid (12408 bp) was obtained by inserting the AleI-StuI fragment of pYes3-T269-GAA100 (48,64) containing our selectable UR-GAA100-A3-TRP1 cassette into the EcoRV site of pJW12. Note, in the pJC_GAA100 plasmid, GAA repeats are in the lagging strand template for replication from the SV40 origin. The pJC_GAA0 (no repeat) plasmid was obtained with the same approach except for the using AleI-StuI fragment from the no-repeat control pYES-TET644 plasmid (48,64). Plasmids were maintained in the *Escherichia coli* SURE strain (Stratagene), and the length of the repeats in isolated plasmids was confirmed by sequencing.

### Oligonucleotides

LNA-ONs and DNA oligonucleotides (ONs) and PNA oligomers were purchased from Eurogentec S.A. The oligonucleotides and oligomers were purified by using reversed phase HPLC, and quality control was performed by using MALDI-TOF mass spectrometry. Control PNA was kindly provided by Prof. Peter Nielsen, Department of Cellular and Molecular Medicine, University of Copenhagen (65).

### Cell culture, transfection, siRNA and ON treatment

HEK-293T (ATCC) were grown in Dulbecco's modified Eagle medium (DMEM, Gibco) supplemented with 10% fetal bovine serum (FBS) and MycoZap^TM^ Plus-CL (Lonza). Cells were transfected with the pJC_GAA100 plasmid by using JetPRIME^®^ (Polyplus-transfection) according to the manufacturer's protocol. Briefly, cells were seeded on day 0 and transfected on day 1 with siRNA. On day 2, cells were co-transfected by siRNA together with pJC_GAA100. siRNA-mediated gene silencing was confirmed by Western blot analysis. Alternatively on day 1, cells were co-transfected with the pJC_GAA100 plasmid and LNA-ON, or PNA, of interest. After additional two days, plasmid DNA was isolated and used for the electrophoretic analysis of replication intermediates (RIs) or transfection into yeast to measure expansion frequencies.

### DNA isolation

Plasmid DNA was recovered 48 h post-transfection by a modified Qiagen Miniprep protocol as described in (44). Briefly, cells were washed with PBS and then resuspended in Qiagen Buffer P1 and lysed in 0.66% sodium dodecyl sulfate, 33 mM Tris–HCl, 6 mM EDTA, 66 μg/ml RNase followed by digestion with 0.5 mg/ml proteinase K for 90 min at 37°C. Samples were subject to brief, 30 s, base extraction with 0.75 ml 0.1 M NaOH, and proteins were precipitated upon addition of Qiagen Buffer P3 (4.2 M Gu-HCl, 0.9 M potassium acetate pH 4.8). Cell debris was pelleted at 29,000*g* for 45 min and supernatant was loaded onto a Qiagen Miniprep spin column. Columns were washed with Qiagen Buffer PB (5 M Gu-HCl, 30% ethanol, adding 10 mM Tris–HCl, pH 6.6) and 0.75 ml Qiagen Buffer PE (10 mM Tris–HCl, pH 7.5, 80% ethanol) and plasmid DNA was eluted using two volumes of 25 μl of Qiagen EB buffer (10 mM Tris-Cl, pH 8.0).

### Yeast transformation

Plasmid DNA from human cells was digested for one hour with 50 DpnI (NEB) units to eliminate unreplicated bacterial DNA, EtOH precipitated, resuspended in TE buffer and transformed into yeast SMY537 strain (*MAT*a, *leu2-Δ1, trp1-Δ63, ura3–52, his3–200*, *bar1::HIS3*, can1::KanMX (64) by the lithium acetate method (66). 5% of each transformation mixture was plated onto SC-Trp plates (synthetic complete, lacking tryptophan), and the remainder 95% onto 5-FOA-Trp (synthetic complete, containing 0.09% 5-FOA and lacking tryptophan) to score for expansions. Colonies on each plate were counted after three days of growth at 30°C. All colonies from 5-FOA-Trp plates were analyzed by single-colony PCR (described below) to verify repeat expansions. Only colonies with PCR-confirmed repeat expansions were tallied in the expansion frequency calculations.

To ensure that expansion events occurred during plasmid replication in human cells and not during yeast transformation, we also established expansion-events baseline by transforming yeast directly with 100 ng of pJC_GAA100 plasmid isolated from *E. coli* without passing through mammalian cells.

### PCR analysis

To verify expansions and to determine their size, individual 5-FOA-resistant yeast colonies were disrupted with Lyticase as described in (4). Subsequent PCR amplification by Phusion Polymerase (ThermoFisher) with UC1 (5′-GGTCCCAATTCTGCAGATATCCATCACAC-3′) and UC6 (5′-GCAAGGAATGGTGCATGCTCGAT-3′) primers flanking the repeat tract was conducted for 35 cycles: of 20 s at 98°C, 2 min at 72°C and 4 min at 72°C. The products were separated into 1.5% agarose gels. PCR product sizes were determined by comparing them with a 50 bp DNA ladder (New England Biolabs) using ImageLab™ software (Bio-Rad).

### Two-dimensional (2D) agarose gel electrophoresis

Plasmid replication intermediates, extracted from human cells as described above, were digested by DpnI, XbaI and BrsGI restriction endonucleases (New England Biolabs), EtOH precipitated and resuspended in TE buffer. The first dimension of electrophoresis was conducted in a 0.4% agarose gel in 1× TBE buffer (89 mM Tris–borate, 2 mM EDTA) at 1 V/cm at room temperature for 19 h. The second dimension was in a 1.3% agarose gel in 1× TBE buffer and was run perpendicular to the first dimension. The dissolved agarose was poured around the excised agarose lane from the first dimension, and electrophoresis was at 5 V/cm in a 4°C cold chamber for nine h in the presence of 0.3 μg/ml ethidium bromide. Gels were washed for 15 min in 0.25 N HCl before an overnight transfer to a charged nylon membrane (Hybond-XL, GE Healthcare) in 0.4 N NaOH. The membrane was hybridized with a ^32^P-labeled radioactive probe corresponding to a 533 bp sequence comprising the *URA3* promoter and part of the CDS of the *URA3* gene. Membranes were washed sequentially twice with washing solution I (2× SSC, 0.1% SDS) at 65°C and twice with washing solution II (0.1 × SSC, 0.1% SDS) at 42°C, exposed on IR-sensitive screens for 1–5 days, and detection was performed on a Typhoon Imager (GE Healthcare). At least three independent transformants were tested for each siRNA knockdown.

### BQQ-OP mediated DNA cleavage of H-DNA forming GAA•TTC repeats in the presence of LNA-ON oligonucleotides

Plasmids pJC-GAA100, pJC_TTC100 and pJC_GAA0 (1 μg) were incubated with 10 mM of either GAA or CTT LNA-ONs (Supplementary Table S3) in a buffer containing 10 mM sodium cacodylate, 100 mM NaCl and 2 mM MgCl_2_ at PH 7.5. As a control, plasmid with no oligonucleotide addition was treated in the same way. The incubations were carried out at 37°C for 16 h. BQQ-OP (1.5 μM) and CuSO_4_ (2.25 μM) were mixed and incubated at room temperature for 15 min. The BQQ-OP/CuSO_4_ mixture was then added to plasmid/oligonucleotide mixture and incubated at room temperature for 45 min. The cleavage reaction was initiated by adding 2 mM 3-mercaptopropionic acid (MPA) and carried out for 3 h at 37°C. DNA samples were purified with miniprep kit (Qiagen), digested with 1U SacI (Thermoscientific) for 1 h at 37°C and separated in 0.7% agarose gel electrophoresis (90 V, 1 h) followed by SYBR-gold (Invitrogen) staining. Gels were analyzed and quantified with VersaDoc MP system (Bio-Rad) and Quantity One software (Bio-Rad), respectively.

### Statistical analysis

At least three independent experiments were conducted for each data point. Statistical analysis was performed via Welch ANOVA test using GraphPad Prism version 8, GraphPad Software, San Diego, California USA.

## RESULTS

### Experimental system to study large-scale expansions of GAA•TTC repeats in cultured human cells

**Figure 1.**
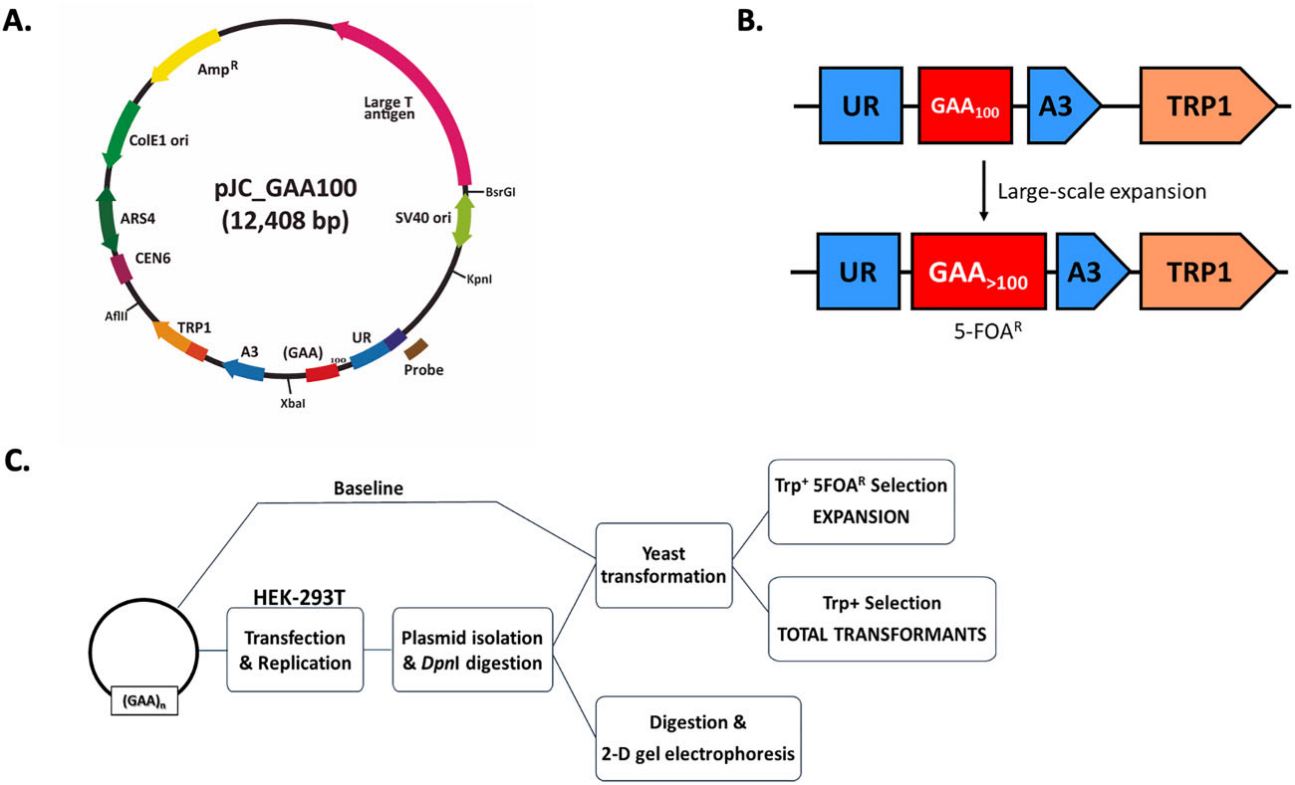
An experimental system to study genome instability and fork stalling caused by GAA•TTC repeats. (**A**) pJC_GAA100 plasmid used in this study. The relative positions of its most relevant features are indicated inside: The centromeric sequence CEN6, the Autonomous Replication Sequence (ARS4), the ColE1 unidirectional origin (ColE1 Ori), the ampicillin resistance gene (Amp^R^), the Large T antigen gene, the SV40 origin of replication (SV40 ori) and the selectable cassette for repeat expansions (*UR-GAA_100_-A3-TRP1*: depicted in B). Outside, the relative positions of sites recognized by specific restriction endonucleases are indicated. (**B**) Schematic of the system to select for repeat expansion in yeast. An artificially split *URA3* gene contains 100 GAA•TTC repeats such that expansion events abrogate splicing and result in resistance to a 5-fluoroorotic acid (5-FOA-r). The addition of more than 10 repeats increased the overall length of the intron beyond the splicing threshold. The selectable cassette was cloned into the pJC_GAA100 plasmid. *TRP1* - an auxotrophic marker for the selection of yeast transformants bearing the plasmids. (**C**) Schematic representation of the assay. Plasmids for the study were transfected into HEK-293T cells, and after culturing them for 48 h, DNA was isolated and digested with DpnI. Repeat expansions were detected upon DNA transformation into yeast. Single-colony PCR was performed for 5-FOA-r clones to confirm expansion events. Expansion frequency was calculated by dividing the number of colonies with repeat expansions by the total number of TRP^+^ transformants. To calculate background expansion frequency, pJC_GAA100 plasmid isolated from *E. coli* was transformed directly into yeast. To study DNA replication through the repeats, DNA was digested with appropriate restriction enzymes and replication intermediates were analyzed by 2-dimensional (2D) agarose gel electrophoresis.

Our new experimental strategy to study large-scale expansions of GAA•TTC repeats and repeat-mediated fork stalling in cultured human cells is schematically presented in Figure 1. We created a shuttle plasmid (Figure 1A) that encodes Tag for efficient replication from the SV40 origin in human cells, an ARS4-CEN6 module for stable maintenance in yeast, and ColE1 replication origin to conduct cloning in *E. coli*. Importantly, it also contains our well-characterized cassette to detect large-scale repeat expansions, which consists of an artificially split *URA3* reporter gene with the (GAA)_100_•(TTC)_100_ repeat in its intron followed by the *TRP1* gene (*UR*-GAA100-*A3 TRP1* in Figure 1B). The addition of 10 or more repeats within the intron abrogates splicing of the *URA3* reporter, rendering yeast resistant to 5-fluoroorotic acid (5-FOA^r^) (Figure 1B). Note that in our plasmid, the homopurine (GAA)_100_ run is in the lagging strand template for DNA replication from the SV40 origin (Figure 1A), which is the orientation that is most prone for repeat instability (35,42,46,67–72).

This plasmid was first transfected into human HEK-293T cells, where it was allowed to replicate for 48 h. Plasmid DNA was then isolated, treated with the restriction enzyme DpnI to remove unreplicated bacterial plasmid DNA, and transformed into yeast that served as a read-out for repeat expansions occurring in human cells. Yeast transformants were plated onto either synthetic complete medium lacking tryptophan (SC-Trp), or to the SC-Trp medium with 0.09% 5-FOA. As a rule, all colonies on the 5-FOA-containing plates were further analyzed by single-colony PCR to verify repeat expansions (Figure 1C). The latter step was important since besides expansions, mutations and deletions within the *URA3* gene can also result in a 5-FOA-resistant phenotype.

### Characterization and genetic controls of GAA•TTC repeat expansions in human cells

**Figure 2.**
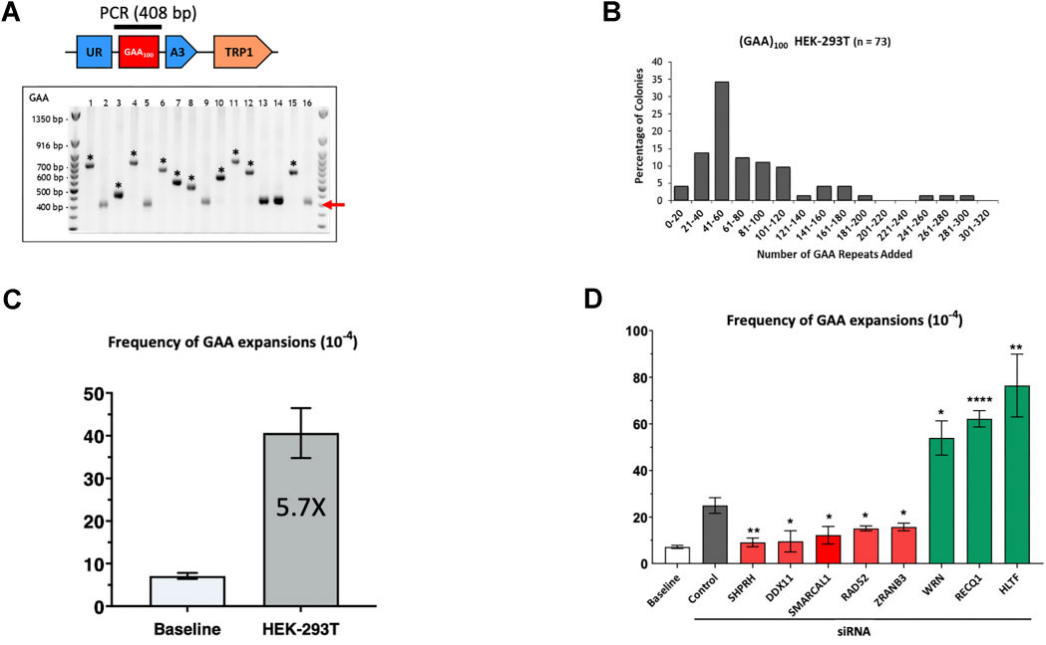
GAA•TTC repeat expansion upon candidate gene knockdown by siRNA. (**A**) Confirmation of GAA•TTC expansions in HEK-293T cells. Repeat lengths in 5-FOA-resistant colonies from independent experiments were analyzed by PCR. On the upper part, a cartoon representation of a cassette with a bar above the GAA•TTC repeats indicates the product of single-colony PCR, used to determine the repeat length. The red arrow points to the length of the PCR product carrying the starting (GAA)_100_•(TTC)100 repeat. ‘*’ marks large-scale GAA•TTC expansions. Only clones with expansions were used to calculate expansion frequencies in (D). (**B**) Distribution of repeats added to the (GAA)_100_•(TTC)_100_ repeat during plasmid replication in HEK-293T cells (dark grey bars). The geometric mean of repeats added is 65 repeats (interquartile range 44.5–100.5). (**C**) Expansion frequencies for pJC_GAA100 plasmid derived from *E. coli* (baseline) and HEK-293T cells. Error bars indicate the standard error of the mean. ‘*’ *P*< 0.05 and ‘**’ indicates a *P*< 0.001 in two-way Welch ANOVA test. (**D**) Expansion frequencies following siRNA gene knockdown in HEK293T cells. The baseline expansion frequency upon yeast transformation by plasmid DNA isolated from *E. coli* (white bar) is shown for the comparison. Error bars indicate the standard error of the mean. Significance compared to the siControl frequency value was determined using a two-way Welch ANOVA test, ‘#’ *P*< 0.05 versus baseline, ‘*’ *P*< 0.05, ‘**’ indicates a *P*< 0.001 versus siControl. See Supplementary Table S1 for details.

Single cell PCR analysis of colonies from 5-FOA plates confirmed that large-scale expansions of GAA•TTC repeats did occur in human cells (Figure 2A). The mean number of added repeats corresponds to 65 (Figure 2B), and we were able to detect up to 300 added repeats when the plasmid replicated in HEK-293T cells. To the best of our knowledge, this is the first system detecting large-scale expansions in mammalian cells after just 48 h in human cell culture.

The frequency of repeat expansions was estimated as the number of 5-FOA-r colonies with PCR-confirmed expansions divided by total number of Trp^+^ transformants using FluCalc calculator (70). These data were compared with the baseline frequency of expansions that either originated in bacteria or in the process of yeast transformation by transforming plasmid DNA isolated from bacteria directly into yeast. Figure 2C shows that the frequency of repeat expansions in HEK-293T significantly (∼6-fold) exceeded the baseline frequency.

To identify proteins involved in large-scale GAA•TTC repeat expansions, we used candidate-gene analysis. We and others have previously identified multiple genes and proteins involved in repeat expansions in various model systems ranging from yeast (42,53) to human cells (43–45). Different proteins were identified in different studies depending on the repeat type, expansion scale, cell type and an organism (reviewed in (1,71–73)). Notably, most of these proteins were the components of DNA replication and repair machineries. We selected 20 candidate genes that encode for proteins involved in DNA replication and post-replication repair. The selected genes were knocked down using pooled siRNAs (Supplementary Figure S1A, B), followed by measuring repeat expansion frequency.

Our candidate genes can be divided into several functional groups. The first group of genes—FEN1, TIMELESS and CLASPIN—encodes replication fork components previously implicated in repeat expansions and instability. Flap endonuclease 1 (FEN1), which is required for the flap-removal during Okazaki fragments maturation and is involved in various DNA repair pathways, was shown to prevent expansions of multiple repeats in a yeast experimental system (1), while its role in mammalian cells remains questionable (74,75). TIMELESS (ScTof1) and CLASPIN (ScMrc1) proteins, which are components of the fork–stabilizing complex, were shown to prevent CAG•CTG repeat instability and expansions in yeast and human cells (76,77), as well as GAA•TTC repeat instability in yeast (46,47,77). In our system, however, siRNA knockdown of these proteins did not affect repeat expansion frequency (Supplementary Figure S2A).

The second group of genes—ATR (yMEC1) and ATM (yTEL1)—triggers DNA damage response (DDR) caused by the replication stress. They were previously shown to stabilize CAG•CTG repeats in yeast, mice and human cells (78). siRNA knockdown of those genes in our system did not show a statistically significant effect on GAA•TTC repeat expansions, albeit the depletion of ATM slightly (1.6-fold) increased repeat expansions (Supplementary Figure S2A).

The third group of genes—RAD51, RAD52, BRCA1 and BRCA2—includes the key components of homologous recombination (HR) and DSB-repair (79,80). Homologous recombination has been implicated in both the promotion and suppression of repeat expansions and instability in various experimental systems (81). In our case, knocking down RAD51, BRCA1 or BRCA2 did not affect GAA•TTC repeat expansions (Supplementary Figure S2A). At the same time, knocking down RAD52 significantly decreased GAA•TTC repeat expansions (Figure 2D). Besides canonical HR (82–84), RAD52 is also implicated in the pathway of DSB repair called break-induced replication (BIR) (85,86). BIR, and specifically RAD52, were shown to promote CAG and CGG repeat expansions in yeast and cultured human cells (87,88). POLD3 (yPol32), a small subunit of DNA polymerase δ, is an essential protein required for DNA synthesis during BIR (89). In our system, the depletion of POLD3 did not change the frequency of GAA•TTC repeat expansions, arguing against the role of BIR. Finally, RAD52 was shown to protect reversed replication forks from degradation by exonucleases (90). We believe that this function of RAD52 might be essential for GAA•TTC repeat expansions in our system (see below and Discussion).

The fourth group of genes—HLTF and SHPRH—encodes ubiquitin ligases involved in the poly-ubiquitination of PCNA critical for DNA damage tolerance, specifically fork reversal and template switching (91,92). The yeast homolog of these genes, RAD5, was shown to promote GAA•TTC and ATTCT•AGAAT repeat expansions (46,93). In addition, the helicase activity of HLTF has been implicated in fork reversal (94). The knockdown of HLTF, but not SHPRH, has been shown to elevate CAG repeat expansions several folds in human cells (95). Here we show that HLTF knockdown increases GAA•TTC repeat expansion frequency (Figure 2D), similarly to what was observed for CAG repeats. At the same time, the knockdown of SHPRH dramatically reduced GAA•TTC repeat expansion frequency (Figure 2D).

The fifth group of genes—SMARCAL1 and ZRANB3—encodes SWI/SNF helicases and ATPases, which catalyze replication fork reversal and restart (96–100). Consequently, depletion or inactivation of these proteins hinders the ability of a replication fork to recover from replication stress, particularly by increasing the frequency of double-strand breaks (96,101–103). We were interested in these proteins because of their role in replication fork reversal and restart, the role of which in promoting repeat expansions in various experimental systems has been widely discussed (1,44,104,105). In line with those data, depletion of either SMARCAL1 or ZRANB3 proteins prevented GAA•TTC repeat expansion in our system (Figure 2D).

The sixth group, DDX11, FANCJ, and RTEL1 are members of a family of iron-sulfur–containing DNA helicases (106,107) that prevent replication stress and mediate HR repair by directly interacting with Pol δ (108,109). We were particularly interested in the DDX11 helicase, as we and others have previously shown that it unwinds triplex structures *in vitro* (110,111). Here, we show that the depletion of DDX11 dramatically decreased repeat expansions (Figure 2D). At the same time, depletion of FANCJ or RTEL1 did not affect GAA•TTC repeat expansions (Supplementary Figure S2A).

We then look at two members of the RecQ family DNA helicases: RECQ1 and WRN that were implicated in the replication fork restart. RECQ1 helicase interacts with PCNA, RPA and DNA polymerase δ (112–117), and has a 3′-5′ directed DNA unwinding capacity restoring reversed forks to their original three-armed configuration *in vitro* and *in vivo* (118). WRN helicase is involved in resolving a variety of DNA substrates: three-way junctions, replication forks, flaps, D-loops, bubbles, Holliday junctions, and G-quadruplexes (G4) (119,120). Here, we show that the depletion of both RECQ1 and WRN helicases increases GAA•TTC repeat expansions (Figure 2D). Besides the RecQ family of helicases, DNA2 is also involved in the processing of reversed forks mediating DNA end resection together other proteins (121). In our system, the depletion of DNA2 did not influence repeat expansions (Supplementary Figure S2A).

As for the mismatch repair, the *MLH1* gene is known to be epigenetically silenced by promoter hypermethylation in HEK-293T cells (122). This is not the case, however, in the parental HEK-293 cells. In our system, the frequency of large-scale expansions did not drastically differ between the two cell lines (Supplementary Figure S2B). This argues against the role of MutL complexes in the expansion process in our system, but it does not rule out other components of the MMR machinery (see Discussion).

In summary, out of 20 candidates tested, five proteins, SHPRH, DDX11, SMARCAL1, RAD52 and ZRANB3 appeared to promote GAA•TTC repeat expansions in our experimental human system, while three proteins, HLTF, WRN and RECQ1 appeared to counteract them (Figure 2D and Supplementary Table S1).

### Replication fork progression through the GAA•TTC repeat

**Figure 3.**
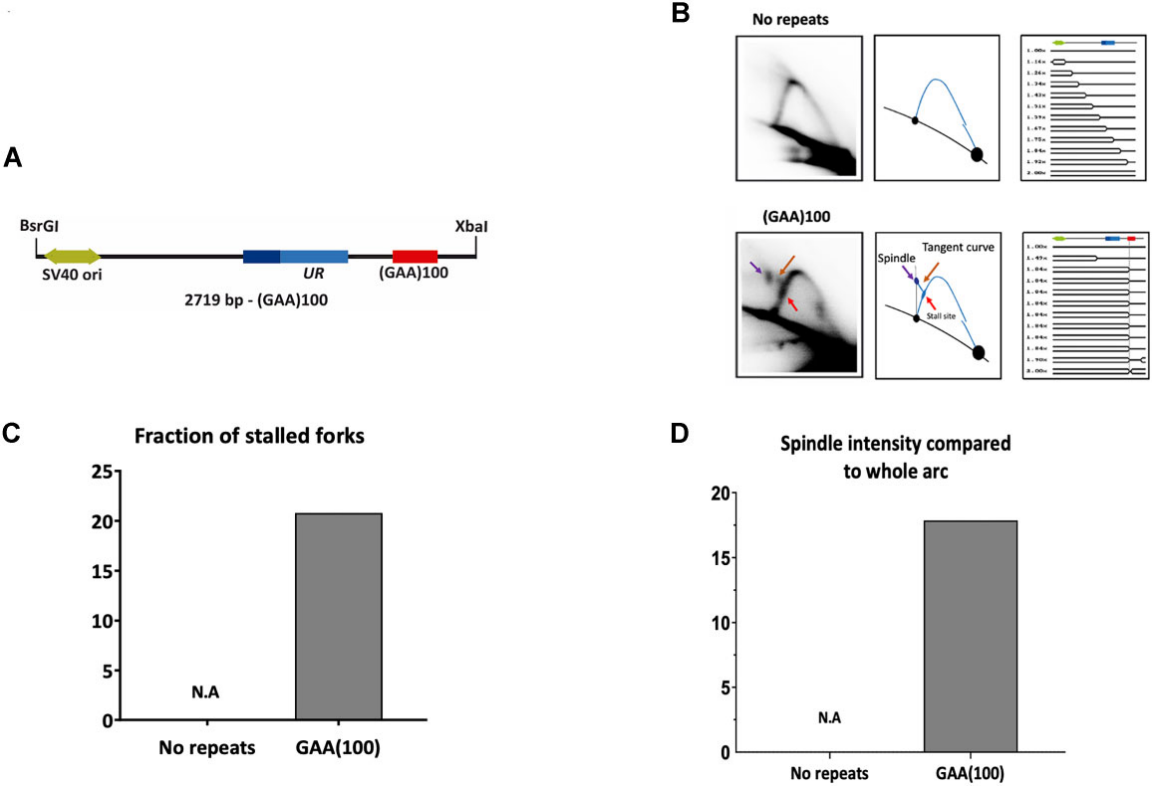
Analysis of pJC_GAA replication in HEK-293T cells by 2D gel electrophoresis. (**A**) Linear map of pJC_GAA100 restriction fragment. (**B**) Representative 2D gels of replication through zero and 100 GAA•TTC repeats are shown in the far-left column with their corresponding interpretative diagrams to their right. DNA was isolated, digested with BsrGI, XbaI, and DpnI, and analyzed by 2D gel electrophoresis. The simulation program 2D gel (123) was used to predict the shape of twelve consecutive replication intermediates (RIs). A linear map is shown on top of each series of RIs, showing the relative positions of SV40 ori (green) UR (blue). The red arrow points to the location of the stall at the (GAA)_100_•(TTC)_100_ repeat. (**C**) Quantification of the fraction of stalled forks. The ratio of radioactivity in the peak area to that corresponding area of a smooth replication arc reflects the extent of replication slowing. Quantification was done with ImageJ (NIH). N.A., non-applicable. (**D**) Quantification of spindle spot intensity compared to the whole arc. The ratio of radioactivity signal at the spindle spot was compared to the radioactivity signal of the whole arc on Image Lab®. N.A., non-applicable.

We and others have previously shown that expanded GAA•TTC repeats stall replication fork progression in yeast and human cells (42–45). Here, to compare repeat expansions with their replication, we analyzed the replication fork progression through the (GAA)_100_•(TTC)_100_ repeat using 2D electrophoretic analysis of replication intermediates. In brief, DNA plasmids with and without the (GAA)_100_•(TTC)_100_ repeat were transfected into HEK-293T cells, replication intermediates were isolated 48 h post-transfection, treated with DpnI to get rid of unreplicated bacterial plasmid DNA, digested by BsrGI and XbaI restriction endonucleases (Figure 3A) and separated by 2D agarose gel electrophoresis followed by Southern hybridization. This digestion scheme deliberately positioned the repeat on the descending part of the replicative Y-arc (Figure 3B).

The simulation program for 2D gels (123) was used to predict the shape of the replication intermediates (RIs) responsible for the patterns observed. The pattern detected for the control plasmid corresponded to that expected for unconstrained replication of the circular plasmid, where the initiation occurs at SV40 origin in a bi-directional manner (simple-Ys's shape) (Figure 3B). However, the repeat-containing plasmid produced a different pattern. In this case, replication would initiate at the SV40 origin bidirectionally, creating a bubble. The leftward (counterclockwise in Figure 1A) moving fork progresses unconstrained, while the rightward (clockwise in Figure 1A) moving fork stalls at GAA•TTC repeats, resulting in the accumulation of simple Y RIs with a mass of ∼1.65×. If this stalling is indefinite, replication will be completed by the leftward replication fork, leading to the accumulation of double-Y RIs migrating above the descending arc. The stalled fork can also reverse, forming chicken feet RIs, which should migrate somewhere in between the stall site and double-Y RIs. The fork reversed at the repeat can be restored, or plasmid replication will be completed by the opposite replication fork (104).

Experimental data shown in Figure 3B clearly demonstrate the presence of the stall site at the expected position on the descending arm and the tangent curve exiting from the stall site linking it to a spindle-shape spot. Based on its electrophoretic mobility, this spindle spot likely represents a mixture of stalled and/or reversed forks, replication of which is completed by the converging fork resulting in double-Y RIs. These results confirm that GAA•TTC repeats strongly stall replication in HEK-293T cells leading to fork reversal and completion of replication by an opposite replication fork in agreement with (44). Note that previously, we and others analyzed GAA•TTC-mediated stall sites on the ascending portion of the Y-arc. This setting makes it somewhat difficult to quantitatively analyze intermediates at different stages of fork reversal, as they can migrate closely to the ascending arc (compare Figures 3B and Supplementary Figure S3).

To quantify our results, we first normalized the signal at the stall site on the descending arm to the signal of the replication arc underneath. This quantification showed that ∼20% of all replication forks stall at the (GAA)_100_•(TTC)_100_ repeat, while no stalling at this position was observed for the no-repeat control plasmid (Figure 3C). The spindle-shaped spot was quantified by comparing its intensity to the rest of the Y-arc (Supplementary Figure S4). It accounted for a ∼17% of RIs in the repeat-containing plasmid and it was not present at all in for the no-repeat control plasmid (Figure 3D).

**Figure 4.**
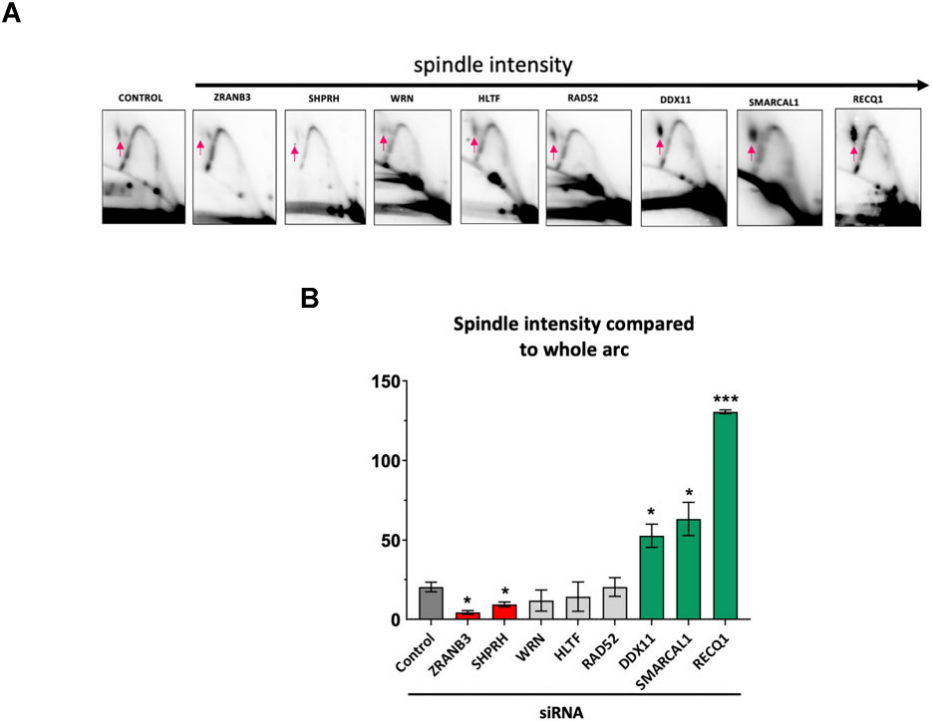
Analysis of replication through GAA•TTC repeats by two-dimensional (2D) agarose gel electrophoresis upon candidate gene knockdown by siRNA. (**A**) Representative 2D gels of replication through the (GAA)_100_•(TTC)_100_ repeats in HEK-293T cells. DNA was isolated, digested with BsrG1, DpnI and XbaI, and analyzed by 2D gel. The red arrow points to the location of the stalling at the GAA•TTC repeats. At least three experiments were analyzed for each siRNA treatment. (**B**) Quantification of the spindle spot at the replication fork. The ratio of radioactivity signal at the spindle spot was compared to the radioactivity signal of the whole arc using Image Lab®. Error bars indicate the standard error of the mean. ‘*’ *P*< 0.05, ‘***’ indicates a *P*< 0.0001 versus siControl. See Supplementary Table S2 for details.

We then analyzed whether proteins that affected GAA•TTC repeat expansions in our system (Figure 2D) influenced replication fork progression through this repeat as well. To this end, we used 2D electrophoretic analysis of RIs isolated from cells depleted of RAD52, ZRANB3, SMARCAL1, SHPRH, HLTF, DDX11, WRN and RECQ1 proteins via siRNA (Figure 4A). Note that depletion of several of these proteins changed the shape of the stall site on the descending arc from the well-defined spot observed in the non-treated cells (Figure 4A control) to a more dispersed signal (see ZRANB3, WRN, RECQ1, SMARCAL1 in Figure 4A). This made the comparison of the stall sites between different siRNA treatments somewhat ambiguous. Thus, we decided to quantitate spindle-shaped RIs that were clearly present in every case, which we considered a proxy for termination RIs, in which one of the two forks was reversed at the GAA•TTC repeat, but not properly restored (Figure 4B and Supplementary Table S2). This reasoning is in-line with the data that effective reversal of one fork is a pre-requisite for the opposite fork to reach the termination site (124). The fraction of those RIs was strongly decreased upon SHPRH and ZRANB3 depletion, but increased upon DDX11, SMARCAL1 and RECQ1 depletion.

In sum, five out of the eight proteins that affected repeat expansions in human cells also changed the character of the replication fork progression through the GAA•TTC repeat. This partial correlation points to a link between replication and large-scale expansions of GAA•TTC repeats in human cells.

### LNA-ONs and PNA oligomers bind and disrupt the intramolecular triplex formed by the (GAA)_100_•(TTC)_100_ repeat

We have previously shown that LNA-modified ONs, which are complementary to either the CTT or GAA strand (GAA LNA-ONs and CTT LNA-ONs, respectively) (Supplementary Table S3) interact with expanded GAA•TTC repeats derived from FRDA patient cells. The GAA LNA-ON significantly disrupted and abolished the pyrimidine-motif H-DNA (H-y DNA) present in supercoiled DNA (26) by forming a duplex-invasion complex. On the other hand, the CTT LNA-ON was able to bind either the remaining GAA single strand of the H-DNA forming Watson-Crick hydrogen bonds or the B-DNA duplex, which resulted in the formation of an intermolecular triplex. These studies were performed using a different plasmid where longer GAA•TTC repeats and additional *FXN* derived flanking sequences where included.

**Figure 5.**
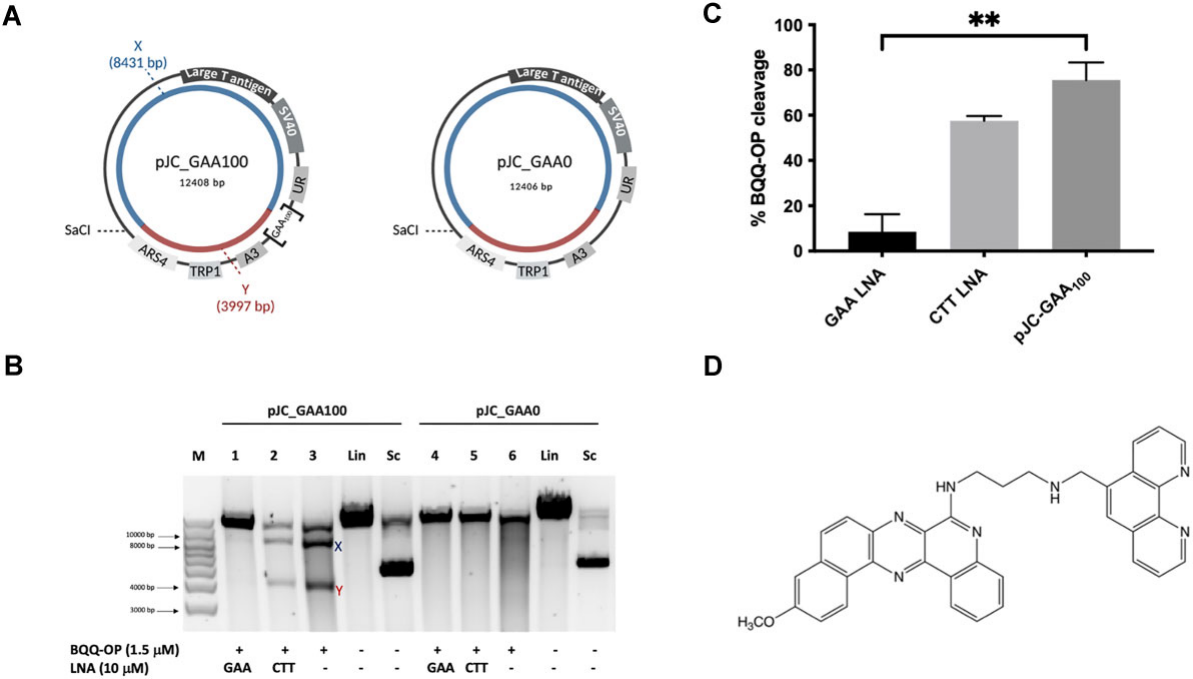
BQQ-OP mediated DNA cleavage of the (GAA)_100_•(TTC)_100_ repeat in the presence of LNA oligomers. (**A**) Schematic illustration of pJC-GAA100 and pJC-GAA0. The H-DNA forming site in pJC-GAA100 is indicated as [GAA_100_]; there is no H-DNA forming site in pJC-GAA0 plasmid. The two DNA fragments generated triplex cleavage by BQQ-OP (benzoquinoquinoxaline 1,10-ortho-phenanthroline) followed by unique site restriction digestion are indicated as X (8431 bp) and Y (3997 bp) in pJC-GAA•TTC and the same reaction would result in a linearized fragment only in pJC-GAA0. (**B**) Representative agarose gel for pJC-GAA100 and pJC-GAA0 plasmids incubated with 10 μM GAA (LNA-DNA) mixmer (lanes 1 and 4 respectively), or CTT (LNA-DNA) mixmer (lanes 2 and 5 respectively), or in the absence of LNAs (lanes 3 and 6 respectively). BQQ-OP-mediated triplex specific cleavage of pJC-GAA100 and pJC-GAA0 was performed in the presence of Cu^2+^ and 3-mercaptopropionic acid (MPA) followed by unique site restriction digestion with SacI. As controls, supercoiled (Sc) and linearized (Lin) variants of both plasmids and molecular weight DNA ladder (M) are shown. (**C**) Graph showing the percentage of BQQ-OP-mediated triplex specific cleavage of pJC-GAA100 in the presence of GAA and CTT (LNA-DNA) mixmers or in the absence of LNA-DNA mixmers (pJC-GAA100). The values represent the ratio between the intensity of DNA double strand cleavage (X + Y) to the total band intensity of the particular lane and are shown as mean with S.D. (*n* = 2). No cleavage was obtained in pJC-GAA0 and not included in the graph. ** indicate P ≤ 0.01 compared to the plasmid in the absence of LNA oligomers. (**D**) Chemical structure of benzoquinoquinoxaline 1,10-*ortho*-phenanthroline (BQQ-OP).

To assess the ability of the (GAA)_100_•(TTC)_100_ repeat in the pJC_GAA100 plasmid to form a triplex structure we used the BQQ-OP triplex-specific cleavage assay. We have previously reported H-DNA formation at FRDA expanded GAA•TTC repeats using the BQQ-OP triplex-specific cleavage assay (26,28,125). BQQ-OP is a low-molecular weight compound that consists of a benzoquinoquinoxaline derivative (BQQ) conjugated to a 1,10-*ortho*-phenanthroline (OP) (Figure 5D). BQQ-OP recognizes and intercalates into both inter- and intramolecular triplex structures, which produces double-strand DNA breaks specifically at the site of triplex formation. Furthermore, we have used the BQQ-OP assay to examine the sequence-specific binding of LNA-ONs and PNA oligomers to the triplex-forming (GAA)_100_•(TTC)_100_ repeat (Supplementary Table S3).

In the case of H-DNA or intermolecular triplex formation at the (GAA)_100_•(TTC)_100_ repeat DNA sequence in the supercoiled pJC_GAA•TTC plasmid, the BQQ-OP triplex-specific double-strand DNA cleavage followed by a unique site cleavage using the SacI restriction enzyme would produce two DNA fragments (∼8431 and 3997 bp-long), which are marked as X and Y in Figure 5A. On the other hand, treatment of the control plasmid, which lacks the repeat sequence (pJC-GAA0), with BQQ-OP and SacI should only produce a single, 12 406 bp-long linear DNA fragment corresponding to the full-length plasmid cleaved by SacI (Figure 5A). Indeed, the two expected DNA fragments were detected after BQQ-OP treatment of pJC_GAA100 plasmid, confirming triplex formation at the repeat (Figure 5B, lane 3). At the same time, the control plasmid did not show any cleavage by the BQQ-OP (Figure 5B, lane 6).

Incubation of the repeat-containing plasmid with GAA LNA-ON dramatically (∼10-fold) decreased BQQ-OP cleavage (Figure 5B, lane 1). These data show that the GAA LNA-ONs essentially prevented H-DNA formation, in agreement with our previous findings (26,126). On the other hand, CTT LNA-ON only marginally affected the triplex-specific DNA cleavage (Figure 5B, lane 2). It is important to note that BQQ-OP cannot distinguish between an intermolecular triplex, where the CTT LNA-ON acts as the third strand targeting the double-strand DNA repeat and the H-DNA (26,126). The difference between the current and previous CTT LNA-ONs data (26) could be due to the variation in the length of the GAA•TTC repeat and the presence of flanking DNA sequences, which might affect the nature and/or the stability of the triplex.

We obtained similar results using GAA PNA and CTT PNA oligomers (Supplementary Figure S5).

### LNA-ONs inhibit GAA•TTC repeat expansions occurring during replication in human HEK-293T cells

**Figure 6.**
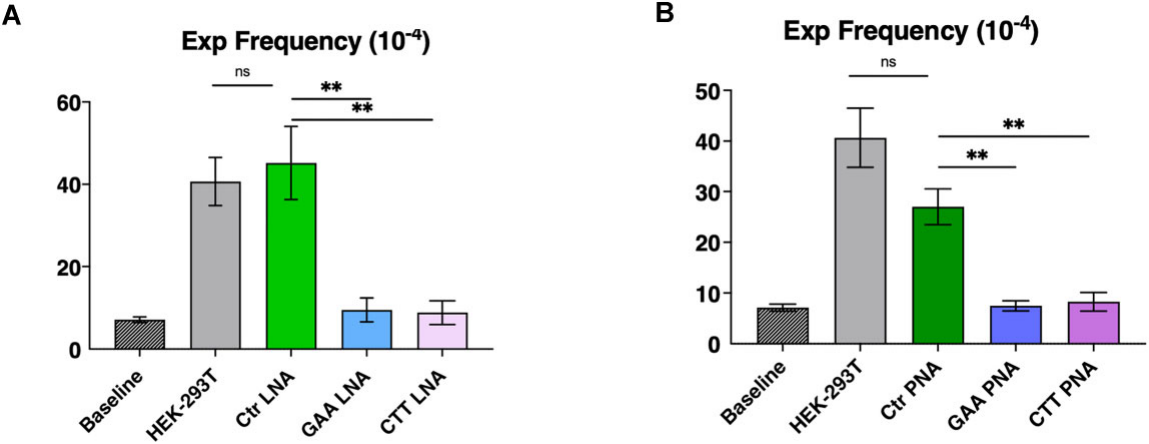
Inhibition of GAA•TTC repeat expansions occurring during replication in human HEK-293T cells by LNA oligonucleotides and PNA oligomers. (**A**) Baseline represents the frequency of the (GAA)_100_•(TTC)_100_ repeat expansion when bacterial pJC_GAA100 plasmid transformed directly into yeast. Other bars in the chart show the frequency of GAA•TTC repeat expansions occurring during plasmid replication in HEK-293T cells in the presence or absence of various LNA-DNA mixmers. Error bars indicate standard error of the mean. Significance relative to the Scramble LNA-DNA mixmer control sequence frequency value was determined using a two-way Welch ANOVA test. ‘*’ *P* ≤ 0.05. (**B**) Baseline represents the frequency of the (GAA)_100_•(TTC)_100_ repeat expansion upon bacterial pJC_GAA100 plasmid transformed directly into yeast. Other columns show the frequency of GAA•TTC repeat expansions that occurred during plasmid replication in HEK-293T cells in the presence or absence of various PNAs. Error bars indicate standard error of the mean. Significance relative to the Scramble PNA sequence frequency value was determined using a two-way Welch ANOVA test. ‘*’ *P* ≤ 0.005.

As discussed above, the frequency of the (GAA)_100_•(TTC)_100_ repeat expansions increased ∼6-fold over the baseline upon plasmid replication in HEK-293T cells (*P* = 0.0043). Here we examined the effect of the chemically modified oligonucleotides, which we previously reported to affect H-DNA formation, on repeat expansions in human cells. To this end, HEK-293T cells were transfected with the pJC_GAA100 plasmid preincubated with either the GAA LNA-ON or CTT LNA-ON (Supplementary Table S3). Strikingly, both oligonucleotides reduced the frequency of repeat expansions essentially down to the baseline level, whereas a control oligonucleotide, which does not bind to the target sequence, had no effect (Figure 6A). We conclude, therefore, that repeat-specific LNA–DNA mixmer oligonucleotides prevent repeat expansions in human cells.

### Inhibition of GAA•TTC repeat expansions in HEK-293T cells by PNA oligomers

To examine whether binding of another class of modified oligomers to the (GAA)_100_•(TTC)_100_ repeat would also affect its expansion frequency in human cells, we studied peptide nucleic acid (PNA) oligomers designed to bind this repeat. PNAs are DNA mimic oligomers with a pseudopeptide backbone (65) that are capable of binding and invading dsDNA in a sequence-specific manner and with high affinity owing to the lack of phosphate repulsion (127). We have previously studied the molecular interaction and binding mode of GAA - and CTT- PNAs (Supplementary Table S3, GAA-PNA and CTT-PNA, respectively) to expanded GAA•TTC repeats in plasmid DNA *in vitro* (26). We have showed that GAA-PNA binds via a duplex-invasion mechanism and completely prevent H-DNA formation by the FRDA expanded repeats. CTT- PNA, on the other hand, formed either a triplex-invasion complex or a Watson-Crick duplex when binding to the complementary polypurine strand of the DNA duplex (26).

Using the same experimental setting as in the previous section, HEK-293T cells carrying the replicating pJC_GAA100 plasmid were incubated with either GAA-PNA or CTT-PNA. This was followed by plasmid DNA isolation and transformation into yeast to detect the frequency of expansion events that accumulated in human cells. Figure 6B shows that similarly to LNA-ONs, both PNA oligomers reduced repeat expansions in human cells to the baseline level, while a control PNA oligomer, which does not bind to the target sequence had only a modest inhibitory effect, which is not statistically significant. We conclude, therefore, that repeat-specific PNAs abolish expansion of GAA•TTC repeats in human cells.

### Dose dependent inhibition of GAA•TTC repeat expansions in human HEK-293T cells by BQQ

**Figure 7.**
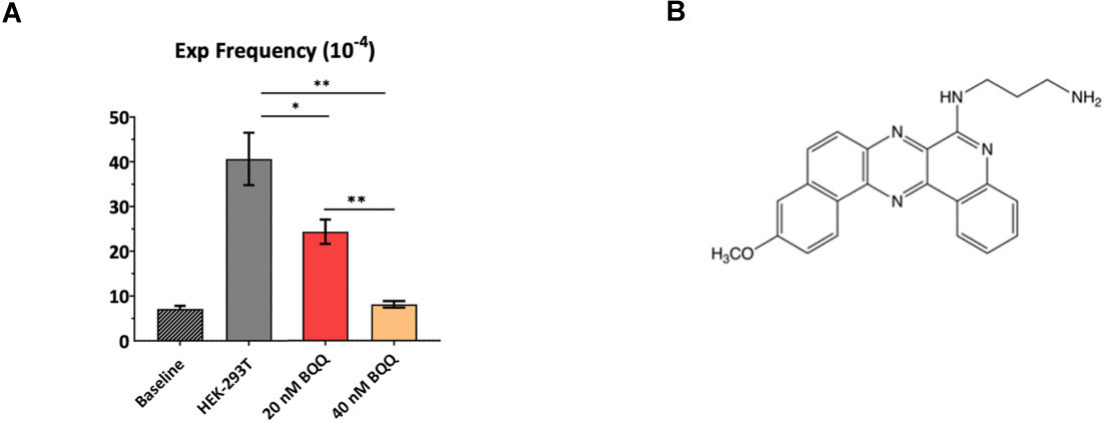
Dose-dependent inhibition of GAA•TTC•TTC repeat expansions in human HEK-293T cells by BQQ. (**A**) Baseline represents the frequency of the (GAA)_100_•(TTC)_100_ repeat expansion when bacterial pJC_GAA100 plasmid was transformed directly into yeast. Other columns show the frequency of GAA•TTC repeat expansions that occurred during plasmid replication in HEK-293T cells at two BQQ (benzoquinoquinoxaline) concentrations (20 and 40 nM). Error bars indicate standard error of the mean. Significance relative to the HEK-293T frequency value was determined using a two-way Welch ANOVA test (for 20 nM BQQ-OP concentration *P* value is ≤ 0.05 for 40 nM, *P* ≤ 0.005). (**B**) Chemical structure of Benzoquinoquinoxaline derivative (BQQ).

Our genetic analysis showed that DDX11 helicase is needed for GAA•TTC repeat expansions in human cells (Figure 2D). Since this helicase is known to unwind a triplex structure formed by the GAA•TTC repeat *in vitro* (111), we reasoned that extra-stabilization of triplex DNA by chemical compounds might prevent expansions as well. To test this hypothesis, we employed the benzoquinoquinoxaline derivative (BQQ) shown in Figure 7B. We have previously reported on the efficiency of this heterocyclic compound to bind DNA polypurine•polypyrimidine sequences once they form an inter- or intramolecular triplexes. BQQ intercalates in the triplex structures with its aminopropyl side chain positioned in the minor groove (128,129). BQQ has also been used to demonstrate the effect of H-DNA formation on transcription in a reporter model in bacteria (130) as well as triplex formation on a genomic level in mammalian cells (111) and its dissociation by the DDX11(ChlR1) helicase.

We treated HEK-293T cells transfected with the pJC_GAA•TTC100 plasmid with BQQ at different concentrations. Figure 7A shows that BQQ treatment significantly decreased the frequency of the GAA•TTC repeat expansion as compared to untreated cells. The BQQ inhibitory effect was dose-dependent: ∼2-fold at 20 nM, and down to the baseline level at 40 nM. We conclude, therefore, that strong BQQ-mediated stabilization of the triplex formed by the (GAA)_100_•(TTC)_100_ repeat impedes repeat expansion in human cells.

## DISCUSSION

In this study, we describe an experimental system to study GAA•TTC repeat instability, in which repeat expansions that occurred during replication of the SV40-based plasmid in human cells were detected upon transformation into yeast (Figure 1). Remarkably, we observed that large-scale repeat expansions occurred very efficiently in this system: up to 300 repeats could be added, while the mean number of added repeats was 65 (Figure 2B). Our experimental setting is similar to that previously developed by Lahue *et* al. for studying CAG•CTG repeat instability (59–61) that was tuned to detect mid-scale (10-to-15 trinucleotides) expansions. The only known instance where massive repeat expansions were observed in a mammalian experimental system was a specific transgenic DM1 mouse (131), yet the reasons for big jumps in the number of CTG repeats in these mice remains unclear. Thus, our experimental system is unique in allowing the study of mechanisms and genetic controls of large-scale repeat expansions in human cells.

We and others have previously found that GAA•TTC repeats cause replication fork stalling in various experimental systems, including SV40-based plasmids (42–45). In accord with these observations, we detected repeat-mediated replication fork stalling and reversal in our experimental plasmid (Figure 3). This data prompted us to investigate various candidate genes that affect DNA replication and post-replicative repair, and/or were previously implicated in repeat expansions in different experimental systems. Altogether, we analyzed twenty candidates and found that siRNA depletion of eight candidates significantly affected the frequency of repeat expansions (Figure 2D). Strikingly, seven of these candidate genes (SHPRH, RAD52, RECQ1, SMARCAL1, WRN, HLTF and ZRANB3) were previously implicated in replication fork reversal/restoration (97–100,118–120,132–141), while the remaining DDX11 is a DNA-helicase unwinding DNA triplex structures *in vitro* (110,111).

We then looked at changes in the replication fork progression through the GAA•TTC repeat upon depletion of the proteins encoded by these eight candidate genes. Inactivation of SHPRH and ZRANB3 decreased, while DDX11, SMARCAL1 and RECQ1 depletion increased the intensity of termination RIs, in which one of the two forks is reversed at the GAA•TTC repeat, but not properly restored. While this is only a partial correlation, a combination of genetic and biochemical data point to the role of the replication fork stalling, reversal, and restart in GAA•TTC repeat expansions during plasmid replication in human cells.

The instability of FRDA GAA•TTC repeats and their role in the disease progression was linked to their ability to form triple-helix H-DNA conformation (reviewed in (142)). This structure is formed when DNA strands in half of the repeat dissociate, and one of them (either polypurine (R) or polypyrimidine (Y)) folds back to form Hoogsteen or reverse-Hoogsteen hydrogen bonds with the remaining duplex, while the complementary strand remains single-stranded (25,31,143). Formation of both H-y and H-r triplex was reported for GAA•TTC repeats at various ambient conditions in supercoiled DNA (25,28,144,145). Here using the BQQ-OP triplex-specific cleavage assay, we confirmed that the (GAA)_100_•(TTC)_100_ repeat forms an H-DNA in our supercoiled pJC_GAA100 plasmid *in vitro* (Figure 5). We reasoned, therefore, that if triplex formation by the GAA•TTC repeat is responsible for its instability, chemical compounds that interfere with triplex formation should affect repeat instability.

We, thus, examined the effect of two distinct classes of modified oligonucleotides LNA-ONs and PNA oligomers, on the repeat expansion frequency. PNA oligomers or LNA-modified oligonucleotides were previously shown to invade the DNA duplex at FRDA GAA•TTC repeats and completely dissolve the preformed triplex structures (26). Here we confirmed that GAA LNA-ONs and GAA-PNA abolished H-DNA formation. In our previous study, we were able to show that CTT LNA-ONs enhanced the amount of triplex formed in the plasmid with FRDA GAA•TTC repeats due to either binding to the remaining GAA single-strand of the H-DNA (H-y) or to the duplex DNA forming an intermolecular triplex. In both cases, CTT LNA-ON binding would lead to the two DNA fragments produced by the BQQ-OP double-strand cleavage. Although the detected effect in the BQQ-OP *in vitro* assay is lower in the current study, our results are still in agreement with the previously reported findings. On the other hand, CTT-PNA, as we have reported earlier, form a triplex invasion complex with expanded GAA•TTC repeats in supercoiled plasmids, which would not be possible to detect using the BQQ-OP assay analyzed by agarose gel electrophoresis. In all cases, it is important to remember that the triplex structures formed during replication are different from those formed in supercoiled plasmids *in vitro*. Nevertheless, the *in vitro* assay demonstrates the capacity of the LNA-DNA oligonucleotides and PNA oligomers to target the GAA•TTC repeats, either through DNA duplex invasion or triplex formation.

Strikingly, we found that both GAA- and CTT- LNAs and PNAs dramatically decreased the expansion frequency in mammalian cells as compared to control oligomers. These data argue against the role of preformed H-y DNA in repeat instability in our system. Rather, both oligomers could prevent transient triplex formation by the repeat in front of the fork (42) or upon fork reversal. Also, BQQ that is known to stabilize DNA triplex structures, counteracted GAA•TTC repeat expansions in a dose-dependent manner. Altogether, chemical compounds that interact with triplex-forming repeat sequences appeared to preclude repeat expansions.

**Figure 8.**
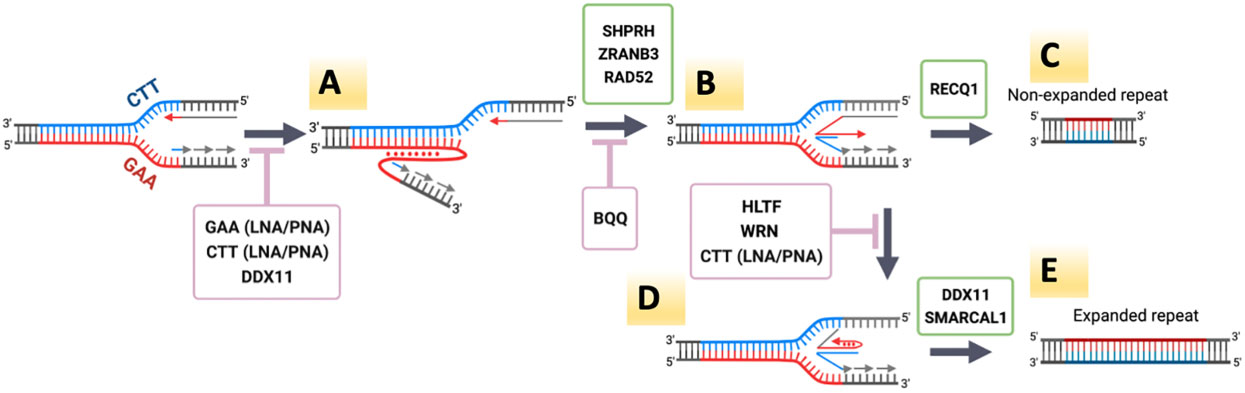
Proposed model for GAA•TTC large-scale expansions. (**A**) The (GAA)_100_•(TTC)_100_ repeat (red – purine strand, blue – pyrimidine strand) can form a triplex during the replication causing fork stalling. This process might be counteracted by repeat-specific LNA-ONs and PNAs, as well as by DDX11 helicase. (**B**) Fork reversal is promoted by SHPRH and ZRANB3, while triplex stabilization by BQQ prevents fork reversal, and the reversed fork is protected from degradation by the RAD52. (**C**) The reversed fork can be restored via an error-free mechanism involving RECQ1 helicase. (**D**) Occasionally, a nascent leading strand can fold back to form a triplex at the tip of reversed fork. This process would be counteracted by HLTF and WRN helicases or in the presence of CTT LNA or PNA. (**E**) Reversed fork capped by the triplex can be restored by an alternative, less precise pathway involving DDX11 and SMARCAL1 helicases, which is accompanied by out of register realignment of repetitive DNA strands, ultimately resulting in expansions.

Genetic control results, electrophoretic analysis of replication intermediates, and triplex stability data altogether led us to speculate on the model of GAA•TTC repeat expansions in our system (Figure 8). During SV40-based replication, the T-antigen serves as a helicase, both the leading and lagging strands are synthesized by DNA Pol δ (146,147), and there is a relatively poor coupling between the helicase (T-antigen) and DNA-polymerase delta (Pol δ) (148), making it prone to fork reversal and restart (148,149). We propose that a triplex transiently formed by the GAA•TTC repeat during DNA replication in front of the fork causes its stalling (Figure 8A), ultimately leading to repeat expansions during replication restart and completion. Triplex formation can be counteracted by LNA-DNA mixmers, PNAs and DDX11 helicase, thus, precluding subsequent steps leading to repeat expansions.

Triplex-mediated fork stalling in the SV40-based plasmids was previously shown to cause fork reversal (44). Our data show that depletion of SHPRH (91), involved in PCNA ubiquitination, and of DNA helicase ZRANB3 reduces repeat expansions and decreases corresponding RIs (Figures 2D and 4B), implicating the two proteins at this step (Figure 8B). Also, RAD52, which is known to protect reversed forks, promotes repeat expansions in our system (90). We suggest, therefore, that fork reversal driven by these proteins is a prerequisite for repeat expansions. In contrast, the presence of triplex stabilizing BQQ makes triplex unwinding and fork reversal difficult, precluding repeat expansions. We are aware that extensive fork reversal usually requires RAD51 protein (150,151). Since we don’t see any effect from the depletion of RAD51 on repeat expansions, we speculate that fork regression in our case might be limited to the repetitive DNA segment.

We show that inactivation of the RECQ1 helicase increases repeat expansion frequency and causes accumulation of RIs corresponding to reversed forks (Figures 2D and 4B). Since RECQ1 is known to efficiently restore reversed forks (100,118,152), we believe that it counteracts addition of extra repeats upon faithful fork restart (Figure 8C). Occasionally, however, a single-stranded overhang of the reversed nascent leading strand can fold back, forming a YR*R triplex at a tip of the reversed fork (Figure 8D). HLTF and WRN helicases are expected to counteract this development, as they are known to bind to single-stranded 3′-overhangs (135,139,140,153–156). Thus, depletion of both helicases would shift the equilibrium towards the reversed fork capped by a triplex. At the same time, by occluding a single-stranded overhangs CTT LNA-ON and PNA would prevent it from folding back to form DNA triplex.

We hypothesize that if DNA triplex at a tip of the reversed fork is not unraveled by the concerted action of the above helicases, an alternative pathway of fork restoration might take place. Depletion of DDX11 and SMARCAL1 decreases GAA•TTC repeat expansions while leading to a significant accumulation of RIs corresponding to reversed forks (Figures 2D and 4B) Both helicases are known to efficiently unwind various non-B DNA secondary structures, including triplex DNA (96,100,108,109). We propose, therefore, that DDX11 and/or SMARCAL1 helicases might drive an alternative, imprecise pathway of fork restoration, during which complementary strands of the repeat could realign out-of-register, ultimately leading to its expansion (Figure 8D).

Alternatively, having one fork reversed at the GAA•TTC repeat intrinsically bears the risk of re-replicating this repeat by the converging fork, which could ultimately lead to repeat expansions. A possibility for re-replication of a DNA segment within the reversed fork was previously proposed in (104).

How do our data and a model compare to other studies of GAA•TTC repeat expansions in mammalian systems? Progressive accumulation of GAA•TTC repeats in a specific HEK-293T cell line that depended on the MutLγ was observed in (55). In this case, only 1-to-2 repeats were added per cell generation. Small-scale GAA•TTC repeat expansions were also observed during propagation of iPSCs cells, and they were impeded by silencing of the MutSα complex (56,157). In the humanized transgenic FRDA mouse, intergenerational expansions of GAA•TTC repeats were inhibited in the presence of a mismatch repair system (158), while somatic expansions were promoted by the MMR (159). In our case, the *MLH1* gene in the HEK-293T cells is epigenetically silenced (122). Furthermore, we see similar frequencies of repeat expansions in HEK293 cell, where *MLH1* is active (Supplementary Figure S2B). On the surface, these data argue against the involvement of MutL complexes in repeat expansions in our system. Clearly, however, more studies with various MMR-deficient cell lines are needed to elucidate the role of other components the MMR system and to decipher the role of MutL in the large-scale GAA•TTC repeat expansions observed by us.

At the same time, our interpretations of the data are in-line with the study that analyzed replication of GAA•TTC repeats in cells from FRDA patients (45). The authors showed that expanded GAA•TTC repeats in iPSCs derived from FRDA patients caused profound replication fork stalling. Notably, GAA•TTC-specific polyamides that were previously shown to alleviate expansion of the GAA•TTC repeats (56) rescued DNA replication as well (45).

Finally, there are evident similarities between our data and the data on smaller-scale CAG•CTG repeat expansions in mammalian cells implicating fork reversal and restart (76,160,161). It is foreseeable that in our case, initial smaller-size expansions of GAA•TTC repeats occurring during fork reversal and restart become larger during subsequent rounds of plasmid replication.

In summary, we developed a first of a kind, genetically tractable experimental system to study large-scale expansions of FRDA GAA•TTC repeats in cultured human cells. Our candidate gene analysis implicates fork reversal and restoration in the process. We believe that this system could be a valuable tool for elucidating the mechanisms of large-scale expansions in humans and for evaluating the efficiency of perspective FRDA drugs targeting the instability of GAA•TTC repeats. Finally, we have showed that LNA-modified oligonucleotides and PNA oligomers targeting the GAA•TTC repeats prevent their expansion holding a promise to be developed as future therapeutics for the treatment of FRDA.

## Supplementary Material

gkad441_Supplemental_File

## Data Availability

The data underlying this article are available in the article and in its online supplementary material.
