# Supplementary material for: Large-scale expansions of Friedreich's ataxia GAA•TTC repeats in an experimental human system: role of DNA replication and prevention by LNA-DNA oligonucleotides and PNA oligomers
Source: Nucleic Acids Res. 2023 May 22;51(16):8532–49. doi: 10.1093/nar/gkad441 (PMC10484681; doi:10.1093/nar/gkad441)

**SUPPLEMENTAL EXPERIMENTAL METHODS**

**Human gene knockdown by siRNAs.**

All ON-TARGETplus SMARTpool^®^ siRNAs (4 individual siRNAs combined) were purchased from Dharmacon:

ATR (L-003201-00-0005), ATM (L-003202-00-0005), BRCA1 (L-003461-00-0005), BRCA2 (L-003462-00-0005), CLSPN (L-005288-00-0005), DDX11 (L-011843-00-0005), FANCJ (L-010587-00-0005) FEN1 (L-010344-00-0005), HLTF (L-006448-00-0005), POLD3(L-026692-01-0005), RAD51 (L-003530-00-0005), RAD52 (L-011760-00-0005), SHPRH (L-007167-00-0005), TIMELESS (L-019488-00-0005), ZRANB3 (L-010025-01-0005), Non-Targeting (D-001810-10-05), RECQ1 (L-013597-00-0005) WRN (L-010378-00-0005).

Antibodies to measure knockdown by Western blots were:

ATR (Cell Signaling Technology mAb #13934), ATM (Cell Signaling Technology mAb #2873), Brca1 (Invitrogen #MA123164), Brca2 (Millipore 05-666), Clspn (Abcam ab94945), Ddx11 (Abcam ab230017), FanJ (Abcam ab180853), Fen1 (Abcam ab17994), Hltf (Bethyl A300-230AM), PolD3 (POLD3 Bethyl Ab PolD3 A301-244A-T), Rad51 (Bioacademia 70-001), Rad52 (Acam ab 124971), Timeless (Abcam ab50943), Shprh (TrueMAB TA501443), Smarcal1 (Abcam6990), Zranb3 (Thermo 23111-1-AP), RECQ1 (Millipore ABC1428), WRN (Abcam ab17987).

**SUPPLEMENTAL DATA**

**Supplementary Table 1. GAA repeat expansion frequencies.**

**Supplementary Table 2. Quantification of spindle intermediates during replication of the GAA100 repeat.**

**Supplemental Table 3. LNA-DNA mixmer oligonucleotides and PNA oligomers used in this study**

**Supplementary Figure 1. Validation of the siRNA-mediated protein depletion by Western Blots.**

**Supplementary Figure 2. Additional GAA repeat expansion frequency analysis.**

**Supplementary Figure 3.** **Analysis of repeat-mediated replication fork stalling on the ascending arm of the Y-arc**.

**Supplementary Figure 4. Analysis of spindle intermediates at the repeat-mediated replication stall on the descending arm of the Y-arc.**

**Supplementary Figure 5. BQQ-OP mediated DNA cleavage of H-DNA forming (GAA)100 repeats in the presence of PNA oligomers**.

**Table S1. GAA repeat expansion frequencies**

**
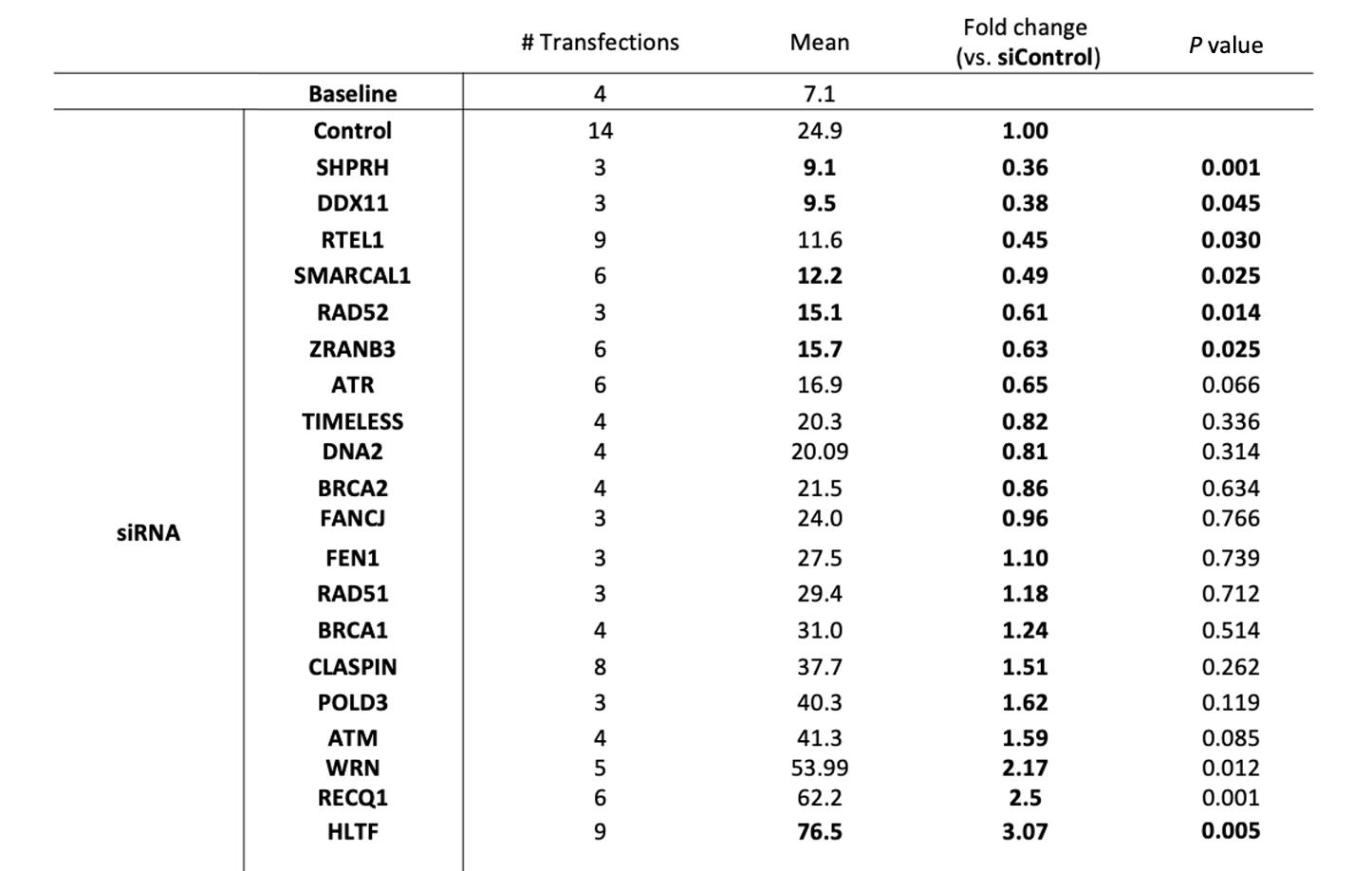
**

**Table S2. Quantification of spindle intermediates during replication of the GAA100 repeat.**

**
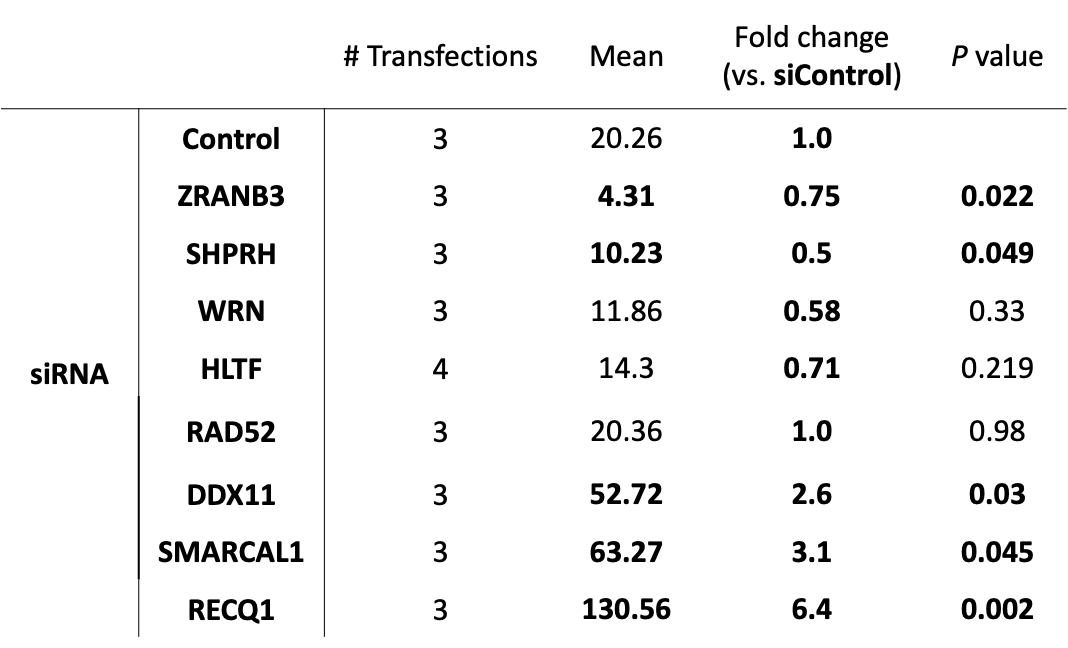
**

**Table S3. LNA-DNA mixmer oligonucleotides and PNA oligomers used in this study**


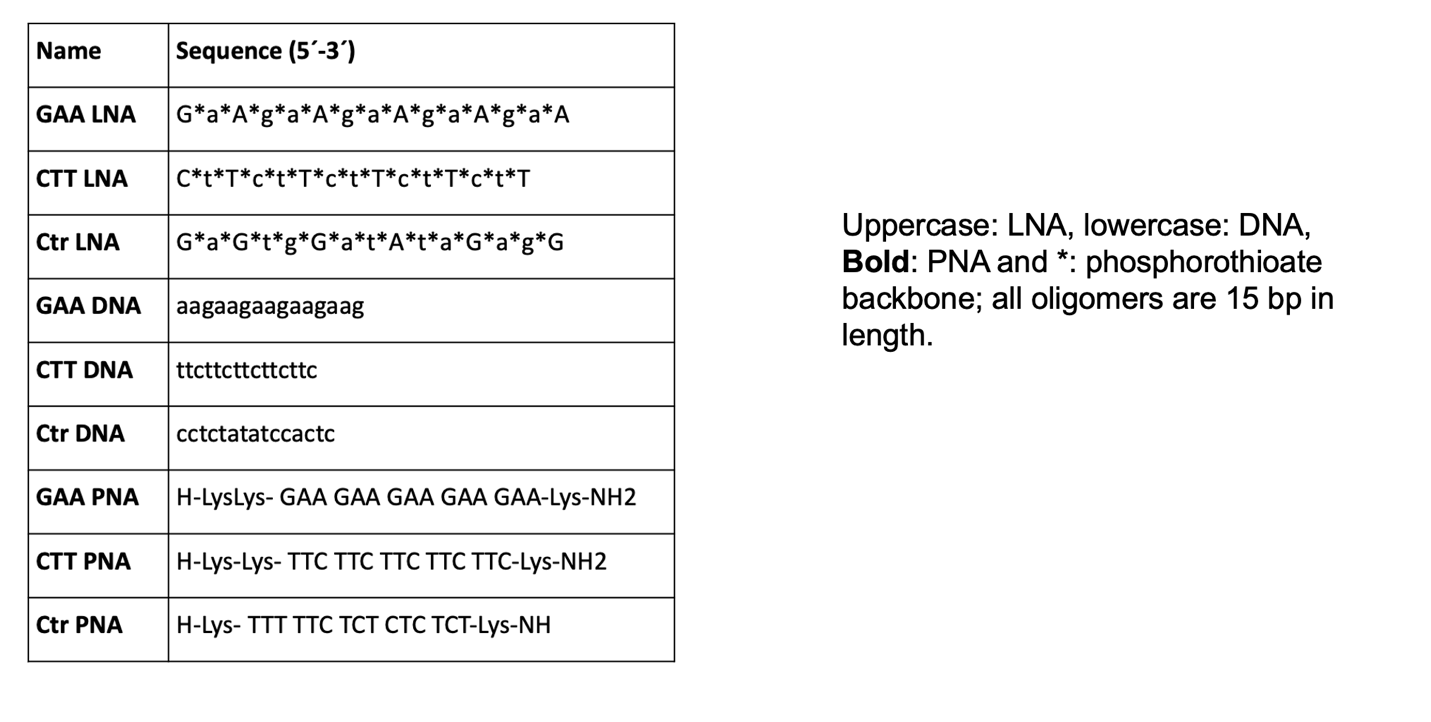


**Figure S1. Validation of the siRNA-mediated protein depletion by Western Blots**: **(A)** Illustrative Western blot showing the effects of siRNAs against the candidate genes studied on the level the corresponding proteins. Protein levels for GAPDH, vinculin and β-actin served as internal controls. **(B)** Quantification of protein levels from several independent experiments.


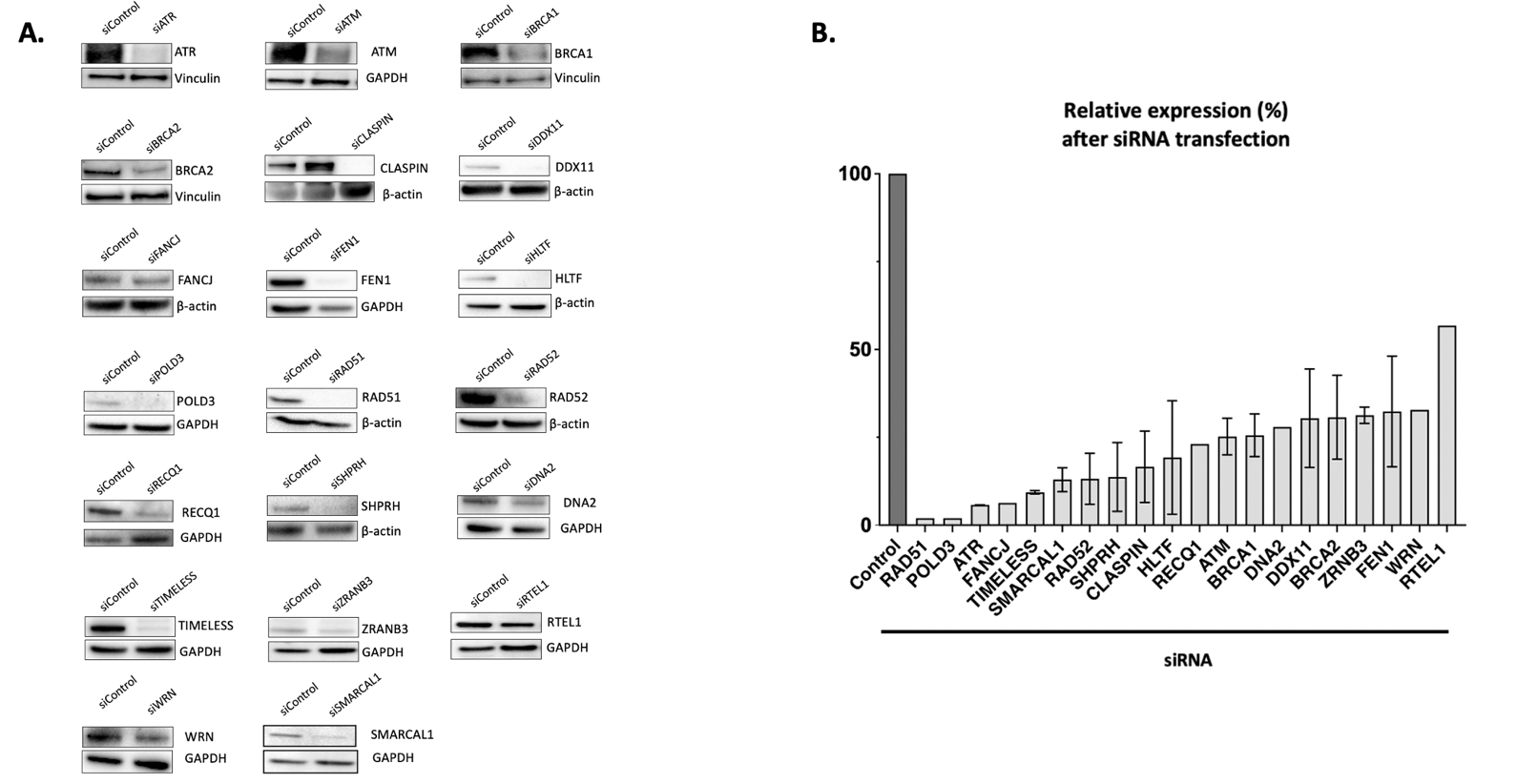


**Figure S2. Additional GAA repeat expansion frequency analysis: (A)** Frequencies of GAA repeat expansions upon candidate gene knockdown by siRNA that show no statistical difference from the control**.** The baseline expansion frequency upon yeast transformation by bacterial plasmid DNA (white bar) is shown for the comparison. Error bars indicate the standard error of the mean. Significance compared to the siControl frequency value was determined using a two-way Welch ANOVA test. See Table S1 for details. **(B)** GAA repeat expansion frequency in HEK293T cells as compared to expansion frequency in HEK293 cells.


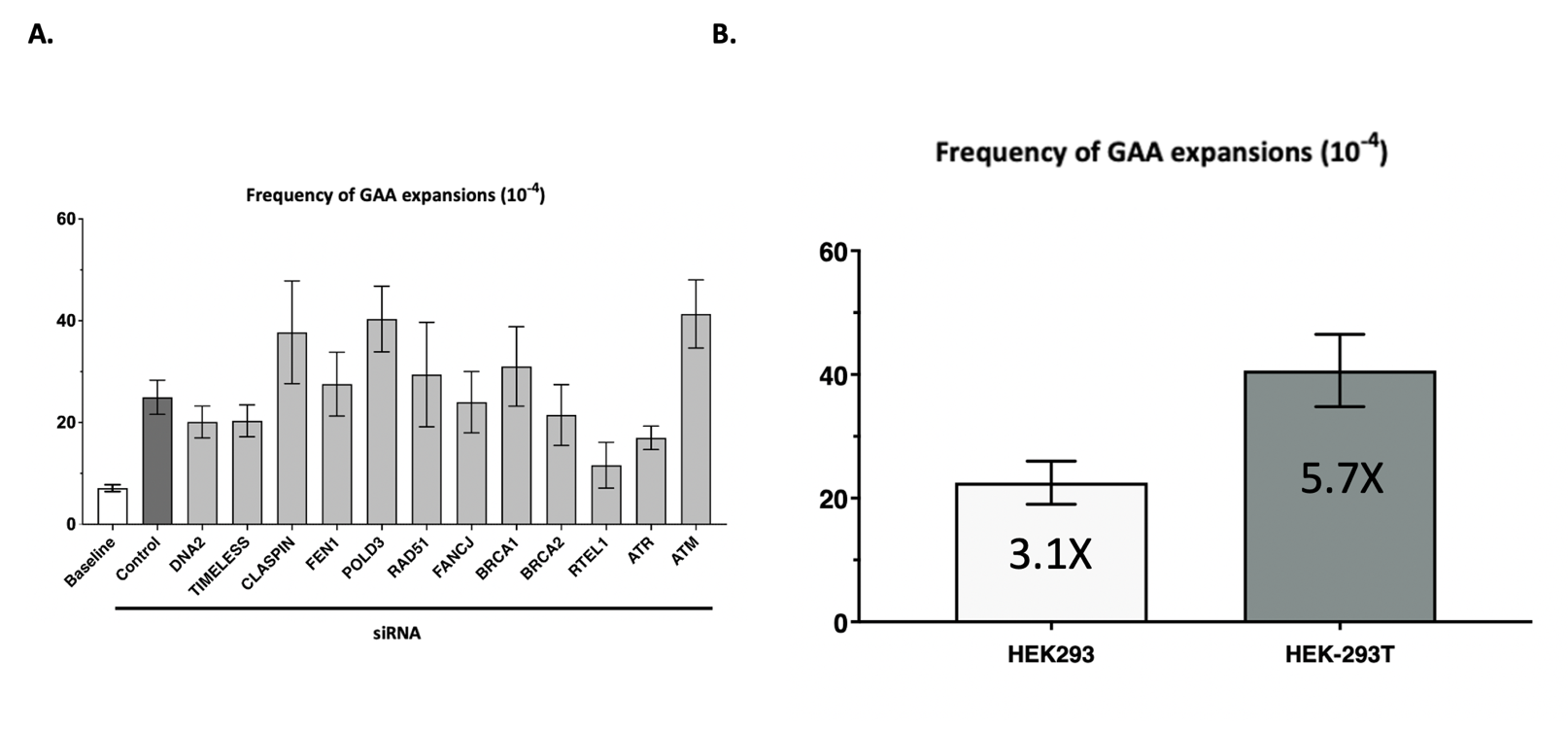


**Figure S3. Analysis of repeat-mediated replication fork stalling on the ascending arm of the Y-arc:** Representative 2D gel of replication through GAA100 repeats is shown in the far-left column with its corresponding interpretative diagram to the right.. The red arrow points to the location of the stall due to the GAA100 repeats with an accompanying marker to show reversed fork migration with Y-arc.


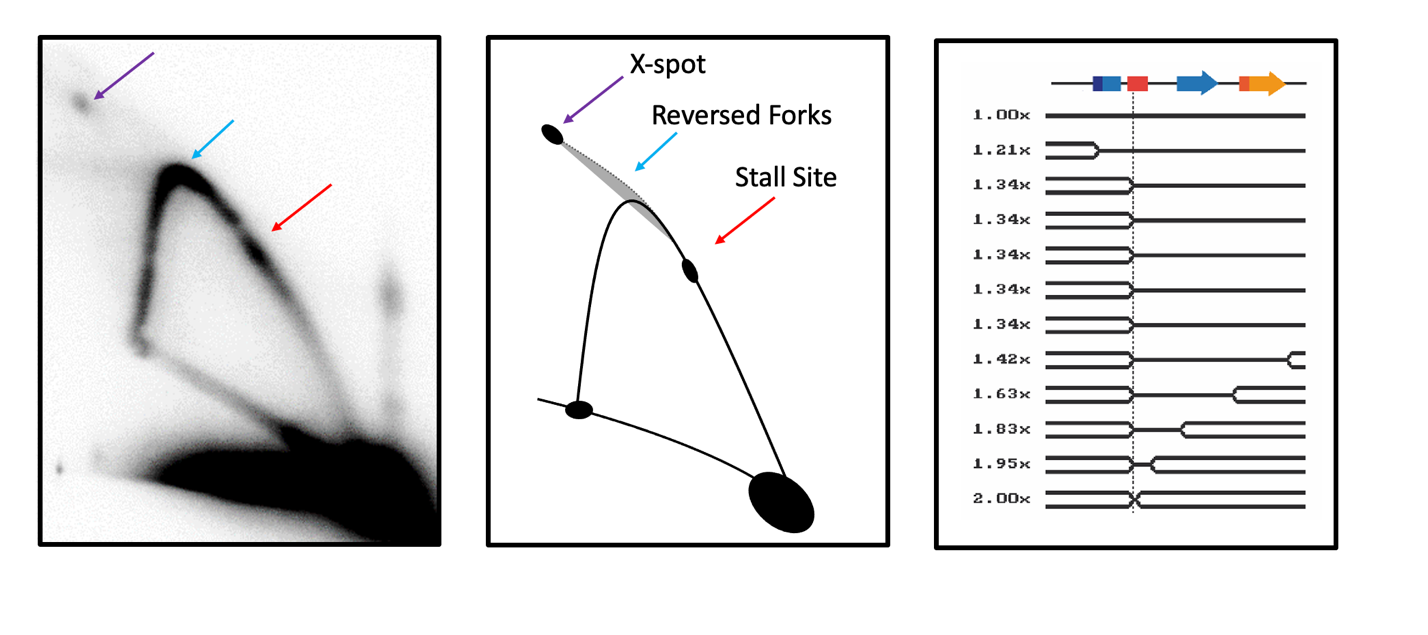


**Figure S4. Analysis of spindle intermediates at the repeat-mediated replication stall on the descending arm of the Y-arc.** Spindle spot quantification was done using ImageLab® as follows. Thee areas were encircled: first around the spindle spot, the second one (B1) to account for the background radioactivity and the third one including total replication Y-arc. Spindle spot strength was estimated by normalizing the signal of the spindle spot to the signal of the whole Y-arc.


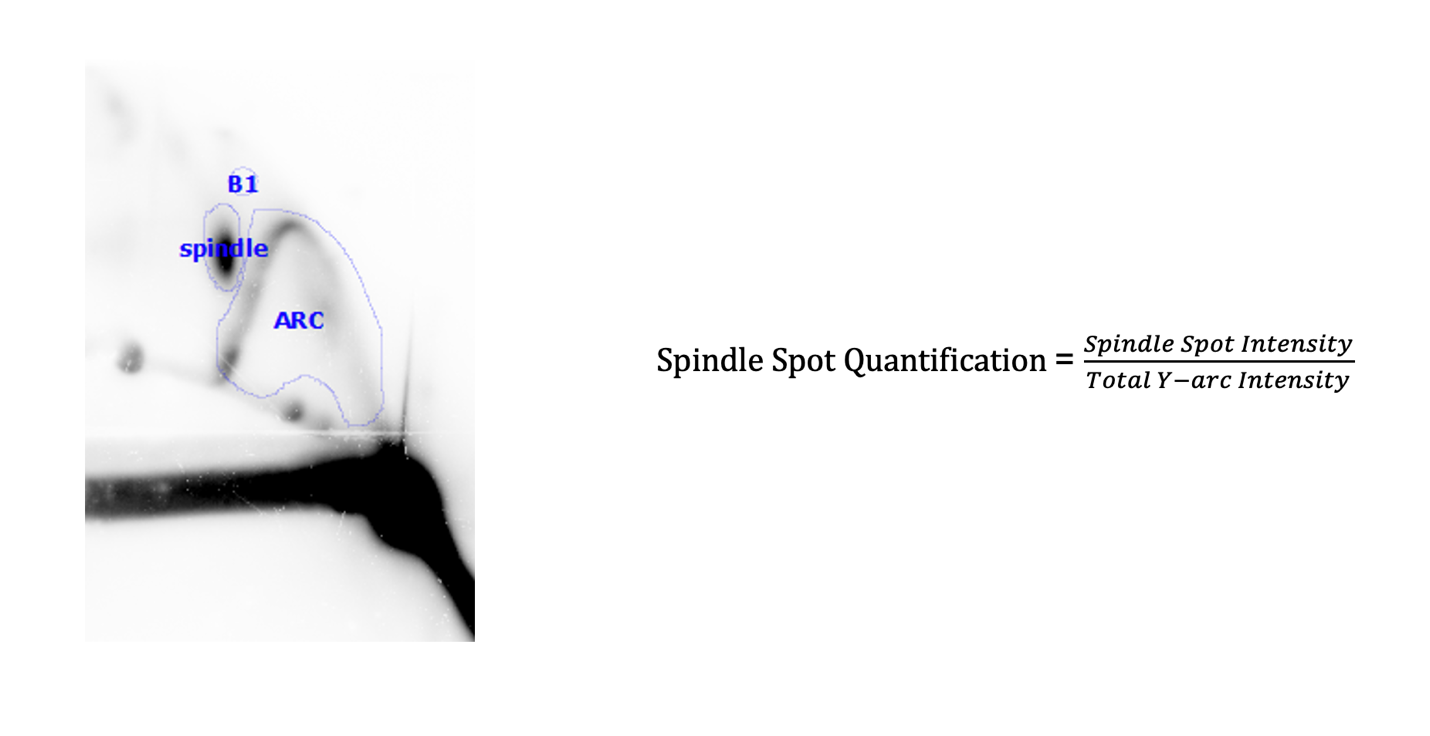


**Figure S5. BQQ-OP mediated DNA cleavage of H-DNA forming (GAA)100 repeats in the presence of PNA oligomers**. Agarose gel analysis for pJC-GAA plasmid incubated with 10 μM GAA PNA oligomer (lanes 1), or CTT PNA oligomer (lanes 2) or in the absence of PNAs (lanes 3). BQQ-OP-mediated triplex-specific cleavage of pJC-GAA was performed in the presence of Cu2+ and 3-mercaptopropionic acid (MPA) followed by unique site restriction digestion with SacI. As controls, supercoiled (Sc) and linearized (Lin) variants of plasmid and molecular weight DNA ladder (M) are shown.


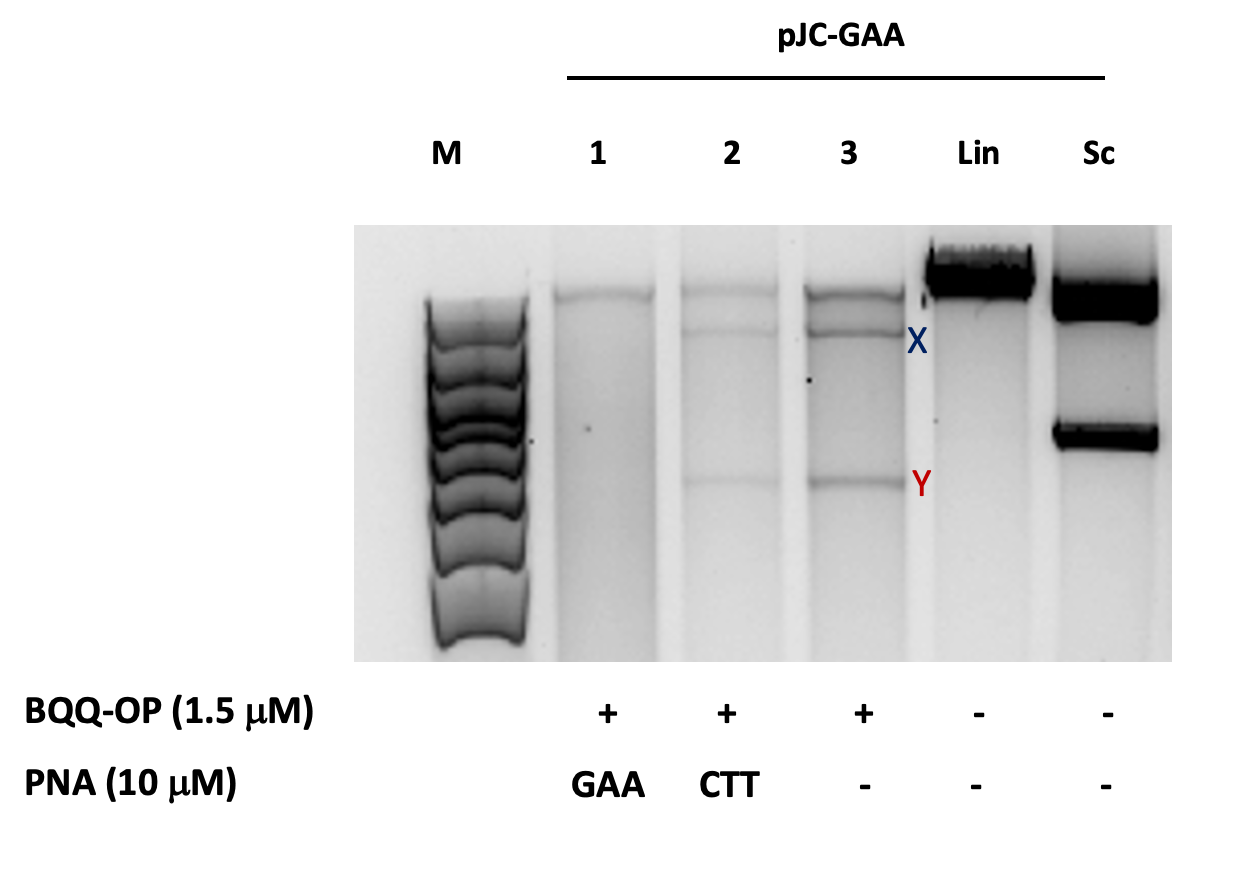

Supplement: gkad441_Supplemental_File [file gkad441_supplemental_file.docx]
